# Dramatic Influence of Ionic Liquid and Ultrasound Irradiation on the Electrophilic Sulfinylation of Aromatic Compounds by Sulfinic Esters

**DOI:** 10.3390/molecules22091458

**Published:** 2017-09-04

**Authors:** Ngoc-Lan Thi Nguyen, Hong-Thom Vo, Fritz Duus, Thi Xuan Thi Luu

**Affiliations:** 1 Department of Organic Chemistry, VNUHCM-University of Science, 227 Nguyen Van Cu St., Dist. 5, 700000 Ho Chi Minh City, Vietnam; lanbmt07@gmail.com (N.-L.T.N.); hongthom2012@gmail.com (H.-T.V.); 2 Department of Science and Environment, Roskilde University, P.O. Box 260, DK-4000 Roskilde, Denmark; fd@ruc.dk

**Keywords:** Friedel-Crafts sulfinylation, sulfinic ester, synthesis of sulfoxide, ultrasound irradiation, chloroaluminate-based ionic liquid

## Abstract

The sulfinylation reaction of aromatic and hetero-aromatic compounds with sulfinic esters as electrophiles has been investigated in different ionic liquids and by means of different Lewis acid salts in order to get moderate to good yields of asymmetrical sulfoxides. Mixtures of 1-butyl-3-methylimidazolium chloride and aluminum chloride were found to be the most efficient and recyclable reaction framework. Ultrasound sonication appeared to be the most useful and green activation method to afford the sulfoxides in yields better than or equivalent to those obtained under the longer-lasting conventional stirring conditions.

## 1. Introduction

Sulfoxides are important intermediates in organosulfur chemistry, especially in the syntheses of drugs and sulfur-containing natural products [1]. Sulfoxides are also used as cardiotonic agents [2], psychotonics [3,4], vasodilators [5], and therapeutic agents for anti-ulcer (proton pump inhibitor) [6,7,8,9,10,11,12], antibacteria, antifungi, or anti-atherosclerosis [13,14,15,16,17], and antihypertension [18].

The importance of sulfoxides in organic syntheses has attracted the attention of chemists to find new methodologies for sulfoxide synthesis. Aliphatic and aromatic sulfoxides have been prepared by the direct and popular oxidation of thioethers [19,20], by indirect reduction of sulfones [21], or by nucleophillic interaction of Grignard reagents on sulfinic esters [22,23], and sulfoxides [24].

Symmetrical aromatic sulfoxides can also be obtained by reaction of arenes with thionyl chloride [25,26], as well as symmetrical/asymmetrical aromatic sulfoxides can be prepared by sulfinylation of aromatic compounds, as described aldready in 1926 [27]. More than thirty years later, alkanesulfinylation and arenesulfinylation of aromatic compounds, (e.g., benzene, azulene/guaiazulene, substituted phenols, and aniline derivatives) with alkanesulfinyl or arenesulfinyl chlorides as electrophiles were investigated in the presence of pyridine [28], anhydrous aluminium chloride [29,30,31], as well as SnCl_4_ or SbCl_5_ [30]; however, owing to the easy decomposition of the sulfinyl halides, harsh reaction conditions were required [28,29,30]. Very recently a replacement of the sulfinyl halides by more stable electrophiles, i.e., sulfinic esters, was introduced in 2011 by Yuste et al. [32]. In connection with the synthesis of (phenylsulfinyl)phenols, the Thia-Fries rearrangement of aryl benzenesulfinate in the presence of aluminum chloride has been studied [33].

The ionic liquid ethylammonium nitrate was synthesized already in the early 1910s [34]. Subsequently, research and development of ionic liquid has concentrated mainly on electrochemical applications since 1948 [35]. In the early 1980s ionic liquids (ILs) attained much more attention owing to their unique chemical and physical properties of non-volatility, non-flammability, thermal stability, and controlled miscibility [35,36]. They have been also used as green solvents in various biphasic ILs-transition metal-catalyzed organic reactions in order to improve catalyst storage, handling, recycling and reuse. The first successful combination of an ionic liquid, dialkylimidazolium chloride, with aluminum chloride used as a Lewis acid catalyst in Friedel-Crafts acylations was reported in 1986 [37].

One of viewpoints on green chemistry, ultrasound irradiation has been regarded as a clean and useful protocol during the last three decades in comparison with traditional activation methods. Many categories of homogeneous or heterogeneous reactions accelerated by ultrasound irradiation have been launched in order to reduce the reaction time, and generate fewer byproducts and a higher yield of the main product [38,39].

**Scheme 1:**
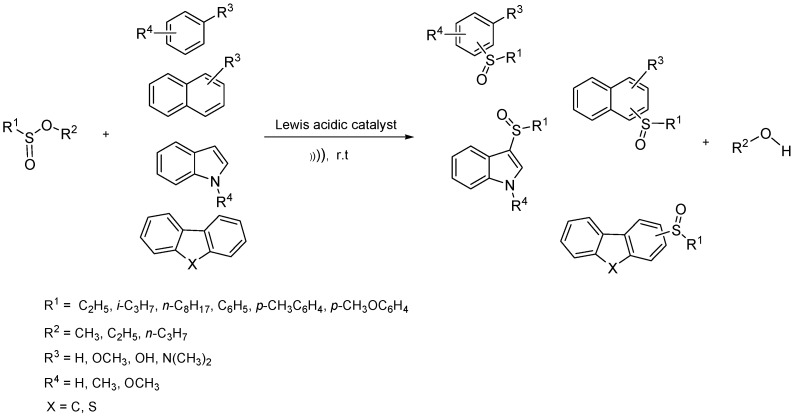
Synthesis of sulfoxides from alkyl alkanesulfinate/arenesulfinate via the sulfinylation reaction.

In this present work, we integrate the advantages of ultrasound irradiation accelerated sulfinylation of arenes and hetero-arenes with stable alkyl alkanesulfinates/arenesulfinates in the presence of different acidic catalysts to produce asymmetrical sulfoxides (Scheme 1). Several Lewis acid salts as well as a task-specific ionic liquid and the mixtures of 1-butyl-3-methyl-imidazolium chloride and different Lewis acid salts with different Lewis acidity were investigated.

## 2. Results

Based on the previous literature and the points of view on green chemistry, a series of Lewis acid salts, for instance bismuth(III) triflate, lanthanide(III) triflate, and copper(II) triflate were used as the acidic catalysts for the solvent-free sulfinylation of 1,3-dimethoxybenzene (our arbitrarily-chosen model substrate) by methyl benzenesulfinate under ultrasound irradiation in order to compare with aluminum chloride catalyst (Entries 1–4, Table 1). The results showed that aluminum chloride efficiently catalyzed the sulfinylation of 1,3-dimethoxybenzene into 2,4-dimethoxy-1-(phenylsulfinyl)benzene under solvent-free as well as solvent reaction condition (Entry 5, Table 1); however, the product selectivity of 2,4-dimethoxy-1-(phenylsulfinyl)benzene reported by GC/MS analyses was achieved 60% from the solvent-free sulfinylation lower than 88% from the solvent sulfinylation owing to severe demethylation of methoxy substituent on aromatic ring. In addition, it is not safe to add anhydrous aluminum chloride directly into the reaction mixture due to the large exotherm.

On the way to find out a green and recyclable acidic catalyst, a series of ionic liquids (Entries 6–12, Table 1), e.g., a task-specific ionic liquid, 1-methyl-3-(4-sulfobutyl)imidazolium hydrogen sulfate, [(HSO_3_)^4^C_4_C_1_Im]HSO_4_, an eutectic mixture of choline chloride and zinc chloride as well as the mixture of [Bmim]Cl and several Lewis acid salts were investigated. The results substantiate our choice of chloroaluminate-based 1-butyl-3-methylimidazole chloride as the most efficient catalyst under ultrasound irradiation. Owing to the mild and efficient catalysis of chloroaluminate-based 1-butyl-3-methylimidazole chloride, demethylation of methoxy substituted group was not found by GC/MS analyses and thin layer chromatography (TLC) during the reaction period.

The composition of chloroaluminate-based ionic liquids is expressed as the apparent AlCl_3_ mole fraction (N) in order to vary their Lewis acidity. They are classified into three types such as base liquids, neutral liquids and acidic liquids [37,40]. Further experiments on the AlCl_3_ mole fraction (Entries 12–14, Table 1) demonstrated that [Bmim]Cl·2AlCl_3_ or [Bmim][Al_2_Cl_7_] (1-butyl-3-methylimmidazole combined with twice molar amount of aluminum chloride, N = 0.67) promoted the sulfinylation of 1,3-dimethoxybenzene to take place most efficiently.

In order to improve the reaction conversion and yield of sulfoxide, the molar ratio of [Bmim]Cl·2AlCl_3_, 1,3-dimethoxybenzene, methyl benzenesulfinate was investigated (Entries 15–17, Table 1). Subsequently, the other reaction factors consisting of reaction temperatures (e.g., room temperature, 50 °C and 60 °C), the reaction time, and the activation methods (e.g., magnetic stirring, ultrasound irradiation and microwave irradiation) were also studied. Consequently, the most efficient procedure for the sulfinylation of 1,3-dimethoxybenzene (1 mmol) with methyl benzenesulfinate (1 mmol) in the appropriate amount of [Bmim]Cl·2AlCl_3_ (1.2 mmol) performed under ultrasound irradiation for one-hour at room temperature was selected (Entry 1, Table 2). Further experiments showed that the highest yield of 2,4-dimethoxy-1-(phenylsulfinyl)benzene was achieved up to 72% after one-hour ultrasound irradiation, while it was 69% after four-hour magnetic stirring. In addition, microwave irradiation is not an efficient method for this reaction owing to the decomposition of reaction mixture at warm temperature (approx. 50 °C).

The optimized conditions were used to investigate the substrate scope of the sulfinylation. Altogether ten aromatic and hetero-aromatic compounds were subjected to [Bmim]Cl·2AlCl_3_-catalysed electrophilic sulfinylation by eight alkyl alkanesulfinate/arenesulfinates under ultrasound irradiation (Table 2 and Table 3).

**Scheme 2:**
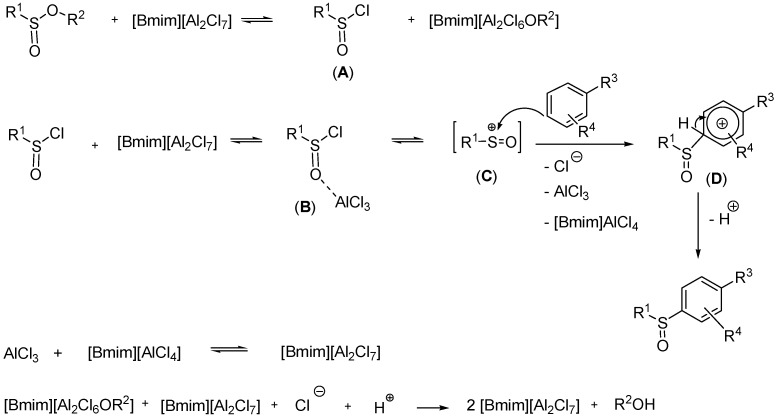
A plausible mechanism for the sulfinylation of aromatic compounds with alkyl arenesulfinates using [Bmim][Al_2_Cl_7_].

The results from altering eight alkyl alkanesulfinate/arenesulfinates used for the sulfinylation of 1,3-dimethoxybenzene demonstrated that the structures of sulfinic esters have influenced the reaction conversion and yield significantly (Table 2). Alkyl arenesulfinates appeared as better electrophiles than alkyl alkanesulfinates. It was, therefore, concluded that the aromatic ring played an important role to stabilize electrons of intermediate in the transition stage (Discussion, Scheme 2).

In the next series of experiments on the influences of substituted benzene or substituted polycyclic benzenoid hydrocarbons, benzene, naphthalene and phenanthrene were selected as model substrates. The results displayed that the reactions of benzene and naphthalene with methyl benzenesulfinate did not occur, while the reaction of phenanthrene worked slowly to obtain a low yield of sulfoxide (4%) under ultrasound irradiation for three hours. Further experiments on the electrophilic sulfinylation of electron rich arenes with methyl benzenesulfinate demonstrated that sulfoxide products were obtained in moderate to good yield after much shorter reaction times (generally 1 h) under ultrasound irradiation, especially most sulfinyl groups were predominantly located at *para* position compared with the former activating substituents on aromatic substrates in order to reduce the steric hindrance (Entries 1–6, Table 3). Contrarily, a sulfinyl group was favorably occupied at the *ortho* position compared with hydroxyl group in order to reach a lower transition free energy and more stable product from the formation of intra-hydrogen bond between hydroxyl group and sulfinyl group (Entry 3, Table 3).

In the continuation of substrate investigation, the reactions of indole derivatives and methyl benzenesulfinate showed that the amount of sulfoxide formed from the sulfinylation of indole was obtained in a lower yield than it from the sulfinylation of 1-methylindole due to a complex formation between indole and [Bmim][Al_2_Cl_7_] (Entries 7 and 8, Table 3).

**Figure 1:**
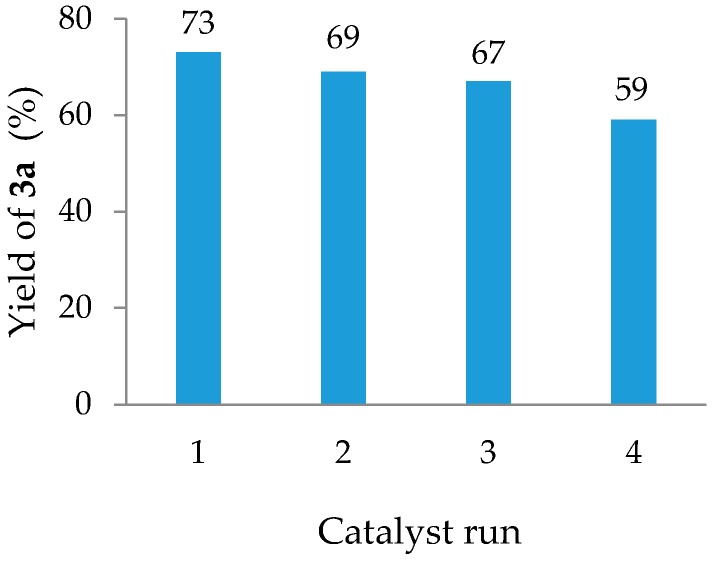
Reusability of [Bmim][Al_2_Cl_7_] (1.2 mmol) in the sulfinylation of 1,3-dimethoxybenzene (1 mmol) with methyl benzenesulfinate (1 mmol) under one-hour of ultrasound irradiation.

With advantages of [Bmim][Al_2_Cl_7_] on enhanced reactivity and mildness, the reusability of [Bmim][Al_2_Cl_7_] was focused on for examination. The [Bmim][Al_2_Cl_7_] collected after separation from the previous reactions was washed with diethyl ether, subsequently evaporated under reduced pressure at 80 °C for 30 min and obtained in 82% of recycled yield. The structure of recovered catalyst at the fourth recycle time was comparable with that of the fresh [Bmim][Al_2_Cl_7_] by ^1^H-NMR spectra. The recycled [Bmim][Al_2_Cl_7_] was used for the sulfinylation of 1,3-dimethoxybezene with methyl benzenesulfinate as that of the optimized experiment presented in Entry 1, Table 2. The catalytic efficiency of [Bmim][Al_2_Cl_7_] did not drop significantly even after three runs of being reused and recycled (Figure 1).

## 3. Discussion

According to several previous investigations on the formation of arenesulfinyl chloride intermediates occurred reversibly from the corresponding alkyl arenesulfinates in the presence of hydrogen chloride and chloride ions [41], arenesulfinyl chlorides (**A**) are obviously generated and preserved under chloroaluminate-based 1-butyl-3-methylimidazole chloride (N = 0.67) owing to a large number of chloride ions. Subsequently, the formation of sulfoxide can be explained by a mechanism similar to that of the Friedel-Crafts sulfonylation of arenes [42], and the sulfenylation of indole [43]. It meant that a complex (**B**) of arenesulfinyl chloride with AlCl_3_ in [Bmim][Al_2_Cl_7_] was formed and then released sulfinyl cation (**C**) which was efficiently stabilized by delocalized electrons of benzene. After that, an intermediate (**D**) could be produced by an eletrophilic sulfinylation of electron rich arenes with sulfinyl cation. Finally, a loss of a proton from (**D**) produced the sulfoxide product (Scheme 2).

## 4. Materials and Methods

### 4.1. Instrumentation

Microwave irradiation was performed by means of a CEM Discover microwave oven produced by CEM Corporation, Matthews, NC, USA. Ultrasound irradiation was performed by means of a BRANSON 1510 ultrasonic bath, operating at 40 kHz with a power output of 70 W. GC/MS analyses were performed on a Hewlett Packard 6890 GC series II, apparatus of MS 5975C with a triple-axis detector equipped with a J and W DB-5MS capillary column (30 m, 0.25 mm i.d., 0.25 µm film thickness) and a Hewlett Packard 7683B autosampler. LC/MS analyses were performed on a micrOTOF-QII-ESI-Qq-TOF (Bruker Daltonics, Bremen, Germany) with UV/VIS and MS detector, the heated capillary of iron trap mass spectrometer was set to 350 °C, reverse column ACE 3C18 (5 µm × 4.6 × 150 mm) and ESI (electrospray ionization): µQTOF Bruker. NMR spectra were recorded on a Brüker 500 NMR spectrometer at 500 MHz (^1^H) and 125 MHz (^13^C).

### 4.2. Chemicals

All commercially available chemicals used were from Aldrich and analyzed for authenticity and purity by GC/MS before being used.

Preparation of commercially unavailable alkyl alkanesulfinates/arenesulfinates: A solution of *N*-bromosuccinimide (6 mmol, 1.067 g) dissolved in methanol (5 mL) and ethyl acetate (1 mL) was added dropwise into the 25 mL two-neck round-bottom flask already containing thiol (3 mmol) in methanol (5 mL), and then 4 mL of methanol was poured additionally up to 14 mL total amount. The flask was placed into an ultrasound bath where the mixture of reactants was exposed to ultrasound irradiation for a specific period of time. Subsequently, the reaction mixture was extracted with dichloromethane (4 × 15 mL). The combined extracts were neutralized with a saturated solution of NaHCO_3_, washed with water until neutral, and then dried by anhydrous Na_2_SO_4_. After removal of the solvent by rotary evaporation, the remaining crude product was analyzed by GC/MS and purified by column chromatography using eluent as a mixture of *n*-hexane and dichloromethane (6:4 *v/v*). The identity and purity of all sulfinic esters obtained were confirmed by ^1^H-NMR, ^13^C-NMR and GC/MS to make sure purity >99% (according to analysis by GC/MS) [44].

Preparation of [Bmim]Cl·2AlCl_3_: An amount of 1-methylimidazole (10 mmol, 1.745 g) and *n*-butyl chloride (11 mmol, 1.018 g) were sequentially added into a 25 mL round-bottom flask assembled with the condenser. The reaction mixture was stirred magnetically with 250 rpm speed at 60–65 °C for 24 h. After cooling down to room temperature, the reaction mixture was washed with diethyl ether (5 × 10 mL) until no trace of *n*-butyl chloride, as well as 1-methylimidazole in a solution of diethyl ether were detected by thin layer chromatography (TLC) and gas chromatography (GC). The other solution containing recyclable [Bmim]Cl was evaporated under reduced pressure at 80 °C for 30 min to afford a light yellow liquid in high yield (88%). The identity and purity of [Bmim]Cl obtained were analyzed by ^1^H-NMR, ^13^C-NMR and LC/MS spectroscopy. In continuation of the preparation of [Bmim]Cl·2AlCl_3_, an amount of aluminum chloride (4 mmol, 0.534 g) was added slowly into a 10 mL round-bottom flask containing [Bmim]Cl (2 mmol, 0.349 g). After that, the reaction mixture were stirred magnetically at room temperature for 12 h. The identity and purity of acidic catalyst, [Bmim]Cl·2AlCl_3_, were checked by ^1^H-NMR and integrated as follows: ^1^H-NMR (500 MHz, D_2_O): δ (ppm) 8.65 (s, 1H), 7.43 (s,1H), 7.38 (s, 1H), 4.14 (t, *J* = 7.0 Hz, 2H), 3.84 (s, 3H), 1.77–1.82 (m, 2H), 1.23–1.30 (m, 2H), 0.87 (t, *J* = 7.5 Hz, 3H).

### 4.3. General Procedure for the Sulfinylation of Aromatic Compound with Sulfinic Ester under Ultrasound Irradiation

An amount of sulfinic ester (1 mmol), [Bmim][Al_2_Cl_7_] (1.2 mmol, 0.530 g) and aromatic compound (1 mmol) were sequentially added into a test-tube (d = 1.5 cm, h = 16 cm) sealed with its cap. The flask was placed into an ultrasound bath where the mixture of reactants was exposed to ultrasound irradiation for a specific period of time. Subsequently, the reaction mixture was extracted with ethyl acetate (3 × 15 mL). The combined extracts were dried by anhydrous Na_2_SO_4_. After the removal of the solvent by rotary evaporation, the residue was purified by column chromatography using eluent as a mixture of *n*-hexane and ethyl acetate.

### 4.4. Spectroscopic Data

The identity and purity of all products reported were confirmed by ^1^H-NMR, ^13^C-NMR, and LC/MS. The unknown NMR spectroscopic data are described below and well-known compounds (**3g** [45], **3i [32]**, **3i**′ [32], and **3k [32]**) have been found compatible with those reported in the literature.

*2,4-Dimethoxy-1-(phenylsulfinyl)benzene* (**3a**). Light brown liquid, ^1^H-NMR (500 MHz, CDCl_3_): δ (ppm) 7.73 (d, *J* = 8.5 Hz, 1H), 7.66 (dd, *J* = 2.0 Hz, *J* = 8.0 Hz, 2H), 7.38–7.41 (m, 3H), 6.62 (dd, *J* = 2.0 Hz, *J* = 8.5 Hz, 1H), 6.39 (d, *J* = 2.0 Hz, 1H), 3.80 (s, 3H), 3.77 (s, 3H). ^13^C-NMR (125 MHz, CDCl_3_): δ (ppm) 163.53, 157.30, 146.01, 130.56, 128.87 (2C), 126.55, 125.04 (2C), 124.90, 105.70, 98.95, 55.69, 55.58. HRMS-ESI: *m/z* [M + H]^+^ calcd. for C_14_H_14_SO_3_, 263.0736; found, 263.0732.

*2,4-Dimethoxy-1-(ethylsulfinyl)benzene* (**3b**). Light brown liquid, ^1^H-NMR (500 MHz, CDCl_3_): δ (ppm) 7.64 (d, *J* = 8.5 Hz, 1H), 6.67 (dd, *J* = 8.5 Hz, *J* = 2.5 Hz, 1H), 6.45 (d, *J* = 2.5 Hz, 1H), 3.84 (s, 3H), 3.83 (s, 3H), 2.97–3.05 (m, 1H), 2.73–2.81 (m, 1H), 1.18 (t, *J* = 7.5 Hz, 3H). ^13^C-NMR (125 MHz, CDCl_3_): δ (ppm) 163.30, 156.36, 127.14, 121.74, 105.20, 98.77, 55.69, 55.61, 46.89, 5.64. HRMS-ESI: *m/z* [M + H]^+^ calcd. for C_10_H_14_SO_3_, 215.0736; found, 215.0758.

*2,4-Dimethoxy-1-(2-propylsulfinyl)benzene* (**3c**). Light brown liquid, ^1^H-NMR (500 MHz, CDCl_3_): δ (ppm) 7.62 (d, *J* = 8.5 Hz, 1H), 6.66 (dd, *J* = 8.5 Hz, *J* = 2.0 Hz, 1H), 6.45 (d, *J* = 2.0 Hz, 1H), 3.84 (s, 3H), 3.83 (s, 3H), 3.04–3.10 (m, 1H), 1.35 (d, *J* = 7.0 Hz, 3H), 1.03 (d, *J* = 6.5 Hz, 3H). ^13^C-NMR (125 MHz, CDCl_3_): δ (ppm) 163.27, 156.80, 127.72, 120.72, 105.17, 98.61, 55.60, 55.51, 51.23, 16.82, 12.72. HRMS-ESI: *m/z* [M + H]^+^ calcd. for C_11_H_16_SO_3_, 229.0893; found, 229.0907.

*2,4-Dimethoxy-1-(octylsulfinyl)benzene* (**3d**). Light brown liquid, ^1^H-NMR (500 MHz, CDCl_3_): δ (ppm) 7.67 (d, *J* = 9.0 Hz, 1H), 6.67 (dd, *J* = 8.5 Hz, *J* = 2.0 Hz, 1H), 6.46 (d, *J* = 2.0 Hz, 1H), 3.84 (s, 6H), 2.94–2.98 (m, 1H), 2.75–2.78 (m, 1H), 1.79–1.82 (m, 1H), 1.58–1.61 (m, 1H), 1.24–1.29 (m, 10H), 0.86 (*t*, *J* = 7.0 Hz, 3H). ^13^C-NMR (125 MHz, CDCl_3_): δ (ppm) 163.31, 156.36, 126.84, 122.46, 105.35, 98.81, 55.72, 55.63, 54.13, 31.75, 29.15, 29.03, 28.68, 22.59, 21.97, 14.04. HRMS-ESI: *m/z* [M + H]^+^ calcd. for C_16_H_26_SO_3_, 299.1675; found, 299.1675.

*2,4-Dimethoxy-1-(p-tolylsulfinyl)benzene* (**3e**). Light brown liquid, ^1^H-NMR (500 MHz, CDCl_3_): δ (ppm) 7.73 (d, *J* = 8.5 Hz, 1H), 7.54 (d, *J* = 8.5 Hz, 2H), 7.21 (d, *J* = 8.0 Hz, 2H), 6.63 (dd, *J* = 2.5 Hz, *J* = 8.5 Hz, 1H), 6.39 (d, *J* = 2.5 Hz, 1H), 3.80 (s, 3H), 3.76 (s, 3H), 2.34 (s, 3H). ^13^C-NMR (125 MHz, CDCl_3_): δ (ppm) 164.05, 157.82, 143.25, 141.64, 130.21 (2C), 127.06, 125.81 (2C), 125.48, 106.19, 99.54, 56.31, 56.20, 21.98. HRMS-ESI: *m*/*z* [M + H]^+^ calcd. for C_15_H_16_SO_3_, 277.0893; found, 277.0892.

*2,4-Dimethoxy-1-((4-methoxyphenyl)sulfinyl)benzene* (**3f**). Light brown liquid, ^1^H-NMR (500 MHz, CDCl_3_): δ (ppm) 7.76 (d, *J* = 8.5 Hz, 1H), 7.58 (dd, *J* = 7.0 Hz, *J* = 2.0 Hz, 2H), 6.91 (dd, *J* =7.0 Hz, *J* = 2.0 Hz, 2H), 6.64 (dd, *J* = 8.5 Hz, *J* = 2.0 Hz, 1H), 6.39 (d, *J* = 2.0 Hz, 1H), 3.81 (s, 3H), 3.80 (s, 3H), 3.74 (s, 3H). ^13^C-NMR (125 MHz, CDCl_3_): δ (ppm) 163.37, 161.60, 157.05, 137.11, 127.21 (2C), 126.24, 124.96, 114.36 (2C), 105.44, 98.95, 55.69, 55.59, 55.42. HRMS-ESI: *m*/*z* [M+H]^+^ calcd. for C_15_H_26_SO_4_, 293.0842; found, 293.0848.

*1,2-Dimethoxy-4-(phenylsulfinyl)benzene* (**3h**). Light brown liquid, ^1^H-NMR (500 MHz, CDCl_3_): δ (ppm) 7.59 (dd, *J* = 1.5 Hz, *J* = 8.0 Hz, 2H), 7.41–7.45 (m, 3H), 7.19 (dd, *J* = 2.0 Hz, *J* = 8.0 Hz, 1H), 7.13 (d, *J* = 2.0 Hz, 1H), 6.89 (d, *J* = 8.5 Hz, 1H), 3.86 (s, 3H), 3.84 (s, 3H). ^13^C-NMR (125 MHz, CDCl_3_): δ (ppm) 151.79, 150.19, 145.97, 137.13, 130.88, 129.30 (2C), 124.70 (2C), 118.99, 111.21, 107.39, 56.22, 56.19. HRMS-ESI: *m/z* [M + H]^+^ calcd. for C_14_H_14_SO_3_, 263.0736; found, 263.0764.

*N,N-Dimethyl-4-(phenylsulfinyl)aniline* (**3j**). White solid, m.p. 130–131 °C [46], ^1^H-NMR (500 MHz, DMSO): δ (ppm) 7.62 (dd, *J* = 1.5 Hz, *J* = 8.0 Hz, 2H), 7.42–7.50 (m, 5H), 6.70 (dd, *J* = 2.0 Hz, *J* = 7.0 Hz, 2H), 3.01 (s, 6H). ^13^C-NMR (125 MHz, DMSO): δ (ppm) 152.54, 146.38, 130.93, 130.30, 129.07 (2C), 127.83 (2C), 124.71 (2C), 112.08 (2C), 40.21 (2C). HRMS-ESI: *m/z* [M + H]^+^ calcd. for C_14_H_15_NSO, 246.0947; found, 246.0946.

*2-Methoxy-1-(phenylsulfinyl)napthalene* (**3l**). White solid, m.p. 100–101 °C [47], ^1^H-NMR (500 MHz, CDCl_3_): δ (ppm) 8.70 (dd, *J* = 8.5 Hz, *J* = 1.0 Hz, 1H), 7.96 (d, *J* = 9.5 Hz, 1H), 7.78 (dt, *J* = 8.0 Hz, *J* = 1.0 Hz, 1H), 7.64 (dd, *J* = 1.0 Hz, *J* = 8.5 Hz, 2H), 7.45–7.49 (m, 1H), 7.35–7.42 (m, 4H), 7.28 (d, *J* = 9.0 Hz, 1H), 3.96 (s, 3H) [47]. ^13^C-NMR (125 MHz, CDCl_3_): δ (ppm) 157.52, 145.26, 134.96, 131.99, 129.72, 129.64, 128.83 (2C), 128.79, 128.21, 124.65, 124.48 (2C), 123.74, 123.26, 113.32, 57.10. MS (C_17_H_14_SO_2_): *m*/*z* = 282[M]^+^, 266, 234, 127, 77.

*1-Methyl-3-(phenylsulfinyl)indole* (**3m**). White solid, m.p. 120–120.5 °C [48], ^1^H-NMR (500 MHz, DMSO): δ (ppm) 8.10 (s, 1H), 7.64 (dd, *J* = 1.5 Hz, *J* = 8.0 Hz, 2H), 7.50–7.54 (m, 3H), 7.46 (tt, *J* = 7.0 Hz, *J* = 1.0 Hz, 1H), 7.25 (dd, *J* = 8.0 Hz, *J* = 1.0 Hz, 1H), 7.22 (td, *J* = 8.0 Hz, *J* = 1.0 Hz, 1H), 7.01 (td, *J* = 8.0 Hz, *J* = 1.0 Hz, 1H), 3.85 (s, 3H) [48].^13^C-NMR (125 MHz, DMSO): δ (ppm) 144.91, 137.56, 134.15, 129.99, 128.99 (2C), 124.39 (2C), 123.58, 122.85, 120.90, 119.05, 115.33, 111.14, 33.02. HRMS-ESI: *m/z* [M + H]^+^ calcd. for C_15_H_13_NSO, 256.0791; found, 256.0782.

*3-(Phenylsulfinyl)indole* (**3n**). White solid, m.p. 126–127 °C [48], ^1^H-NMR (500 MHz, DMSO): δ (ppm) 11.93 (s, 1H), 8.11 (d, *J* = 3.0 Hz, 1H), 7.63–7.66 (m, 2H), 7.50–7.54 (m, 3H), 7.44–7.48 (m, 2H), 7.23 (d, *J* = 8.0 Hz, 1H), 7.15 (td, *J* = 8.0 Hz, *J* = 1.0 Hz, 1H), 6.94–6.97 (m, 1H) [47]. ^13^C-NMR (125 MHz, DMSO): δ (ppm) 144.98, 137.06, 130.81, 129.95, 128.99 (2C), 124.42 (2C), 123.18, 122.85, 120.66, 119.00, 116.33, 112.71. HRMS-ESI: *m/z* [M + H]^+^ calcd. for C_14_H_11_NSO, 242.0634; found, 242.0634.

*2-(Phenylsulfinyl)dibenzo[b,d]thiophene* (**3p**). White solid, ^1^H-NMR (500 MHz, CDCl_3_): δ (ppm) 8.55 (d, *J* = 1.0 Hz, 1H), 8.22–8.24 (m, 1H), 7.90 (d, *J* = 8.0 Hz, 1H), 7.85–7.87 (m 1H), 7.71 (dd, *J* = 8.0 Hz, *J* = 1.5 Hz, 2H), 7.59 (dd, *J* = 8.5 Hz, *J* = 1.5 Hz, 1H), 7.51–7.52 (m, 2H), 7.44–7.47 (m, 3H). ^13^C-NMR (125 MHz, CDCl_3_) and HSQC: δ (ppm) 145.77, 142.46, 141.88, 139.89, 136.21, 134.69, 131.17, 129.43 (2C), 127.63, 124.93 (3C), 123.75, 122.94, 122.66, 122.15, 118.19. HRMS-ESI: *m/z* [M + H]^+^ calcd. for C_18_H_12_S_2_O, 309.0402; found: 309.0403.

*2-(Phenylsulfinyl)-9H-fluorene* (**3q**). White solid, ^1^H-NMR (500 MHz, CDCl_3_): δ (ppm) 7.82–7.85 (m, 2H), 7.78 (d, *J* = 7.0 Hz, 1H), 7.67–7.70 (m, 2H), 7.65 (dd, *J* = 8.0 Hz, *J* = 2.0 Hz, 1H), 7.55 (d, *J* = 7.5 Hz, 1H), 7.41–7.49 (m, 3H), 7.38 (td, *J* = 7.5 Hz, *J* = 1.0 Hz, 1H), 7.35 (td, *J* = 7.5 Hz, *J* = 1.5 Hz, 1H), 3.91 (s, 1H). ^13^C-NMR (125 MHz, CDCl_3_): δ (ppm) 146.03, 145.08, 144.50, 143.86, 143.71, 140.42, 131.06, 129.45 (2C), 128.03, 127.20, 125.34, 124.90 (2C), 124.26, 121.63, 120.67, 120.56, 37.06. HRMS-ESI: *m/z* [M + H]^+^ calcd. for C_19_H_14_SO, 291.0838; found, 291.0839.

## 5. Conclusions

An efficient, highly regio-selective and green synthetic method to prepare sulfoxides has been developed. Sulfinic esters appeared as greener, safer, and more stable electrophiles than sulfinyl chlorides in order to improve the reaction process at room temperature instead of very low temperature. [Bmim]Cl·2AlCl_3_ is found out to be a better acidic catalyst owing to its efficiency, easy operation and recyclability. Moreover, ultrasound irradiation has good effects on the yields of sulfoxide products within a shorter reaction time.

## Figures and Tables

**Table 1:**
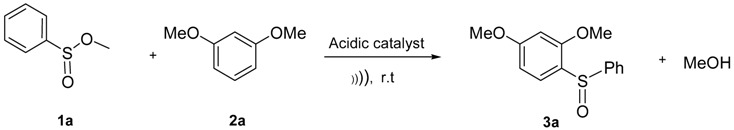
Influence of the nature of the acidic catalyst on the formation of 2,4-dimethoxy-1-(phenylsulfinyl)benzene (**3a**) ^a^.

Entry	Acidic Catalyst (mmol)	Yield ^b^ (%)
1	Bi(OTf)_3_(0.05), solvent-free	3
2	La(OTf)_3_(0.05), solvent-free	-
3	Cu(OTf)_2_(0.05), solvent-free	-
4	AlCl_3_(2.0), solvent-free	16
5	AlCl_3_(2.0) in 1,2-dichloroethane (1 mL)	7
6	[(HSO_3_)^4^C_4_C_1_Im]HSO_4_(1.0)	6
7	[Choline]Cl·3ZnCl_2_ (1.0)	-
8	[Bmim]Cl·2ZnCl_2_ (1.0)	3
9	[Bmim]Cl·2ZnCl_2_ (1.2)	4
10	[Bmim]Cl·2NiCl_2_(1.0)	-
11	[Bmim]Cl·*p*-TSA (2.0)	-
12	[Bmim]Cl·AlCl_3_(1.0)	32
13	[Bmim]Cl·2AlCl_3_(1.0)	55
14	[Bmim]Cl·3AlCl_3_(1.0)	25
15	[Bmim]Cl·2AlCl_3_(1.1)	69
16	[Bmim]Cl·2AlCl_3_(1.2)	72
17	[Bmim]Cl·2AlCl_3_(1.5)	68

^a^ The reactions of 1,3-dimethoxybenzene (1 mmol) with methyl benzenesulfinate (1 mmol) catalyzed by the acidic catalysts were performed under ultrasound irradiation for one hour at room temperature; ^b^ Yield of isolated product.

**Table 2:**
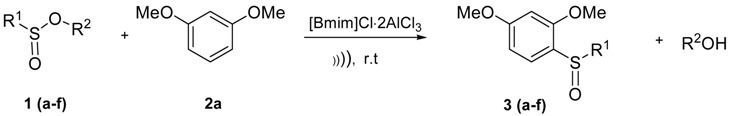
Influence of the nature of the alkyl alkane/arenesulfinates on the yields of asymmetrical sulfoxides ^a^.

Entry	Sulfinic Ester	Product		Time (h)	Yield ^b^ (%)
1	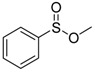	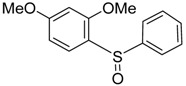	**3a**	1.0 1.5	72 73
2	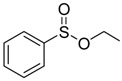			1.0	72
3	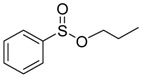			1.0	77
4	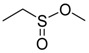	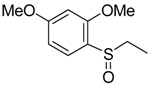	**3b**	1.0 4.0	15 70
5	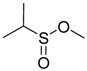	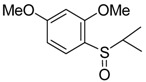	**3c**	1.0 4.5	10 68
6	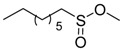	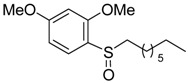	**3d**	1.0 4.0	17 72
7	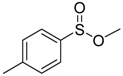	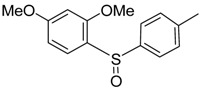	**3e**	1.0	73
8	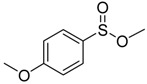	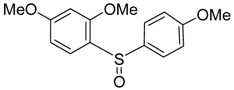	**3f**	1.0	75

^a^ The reactions of 1,3-dimethoxybenzene (1 mmol) with sulfinic ester (1 mmol) catalyzed by [Bmim][Al_2_Cl_7_] (1.2 mmol) were performed under ultrasound irradiation for a specific period of time (in hours) at room temperature; ^b^ Yield of isolated product.

**Table 3:** Influence of the nature of the aromatic compounds on the sulfinylation using methyl benzenesulfinate ^a^.

Entry	Arene	Product		Time (h)	Yield ^b^ (%)
1	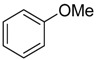	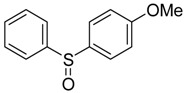	**3g**	1.0	61
2	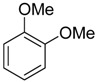	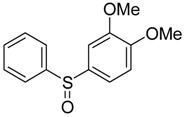	**3h**	1.0	68
3	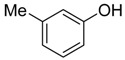	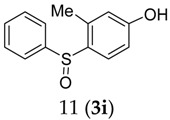	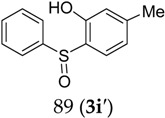	1.0	75
4	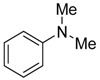	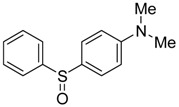	**3j**	1.0	81
5	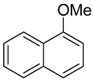	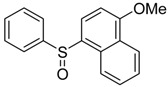	**3k**	2.0	58
6	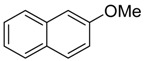	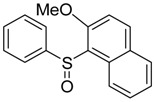	**3l**	3.0	53
7	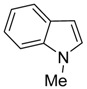	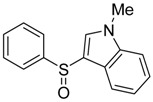	**3m**	0.5	73
8	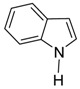	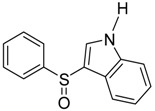	**3n**	0.5	40
9	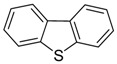	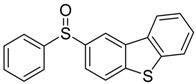	**3p**	8	13
10	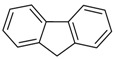	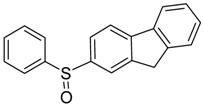	**3q**	8	6

^a^ The reactions of arene (1 mmol) with methyl benzenesulfinate (1 mmol) catalyzed by [Bmim][Al_2_Cl_7_] (1.2 mmol) were performed under ultrasound irradiation for a specific period of time (in hours) at room temperature; ^b^ Yield of isolated product.

## Supplementary Materials

Supplementary materials are available online. ^1^H-NMR and ^13^C-NMR of unknown products.
