# Supplementary material for: Dramatic Influence of Ionic Liquid and Ultrasound Irradiation on the Electrophilic Sulfinylation of Aromatic Compounds by Sulfinic Esters
Source: Molecules. 2017 Sep 4;22(9):1458. doi: 10.3390/molecules22091458 (PMC6151476; doi:10.3390/molecules22091458)

molecules

Supplementary Materials for article:

Dramatic influence of ionic liquid and ultrasound irradiation on the electrophilic sulfinylation of aromatic compounds by sulfinic esters

Ngoc-Lan Thi Nguyen[1], Hong-Thom Vo[1], Fritz Duus[2] and Thi Xuan Thi Luu[1,*]

[1] Department of Organic Chemistry, VNUHCM-University of Science, 227 Nguyen Van Cu St., Dist. 5, Ho Chi Minh City, Vietnam; lanbmt07@gmail.com (N.-L.T.N.), hongthom2012@gmail.com (H.-T.V.)
[2] Department of Science and Environment, Roskilde University, PO Box 260, DK-4000 Roskilde, Denmark; fd@ruc.dk (F.D.)
* Correspondence: ltxthi@hcmus.edu.vn; Tel.: +84-0208-3835-3193

2,4-dimethoxy-1-(phenylsulfinyl)benzene (3a)

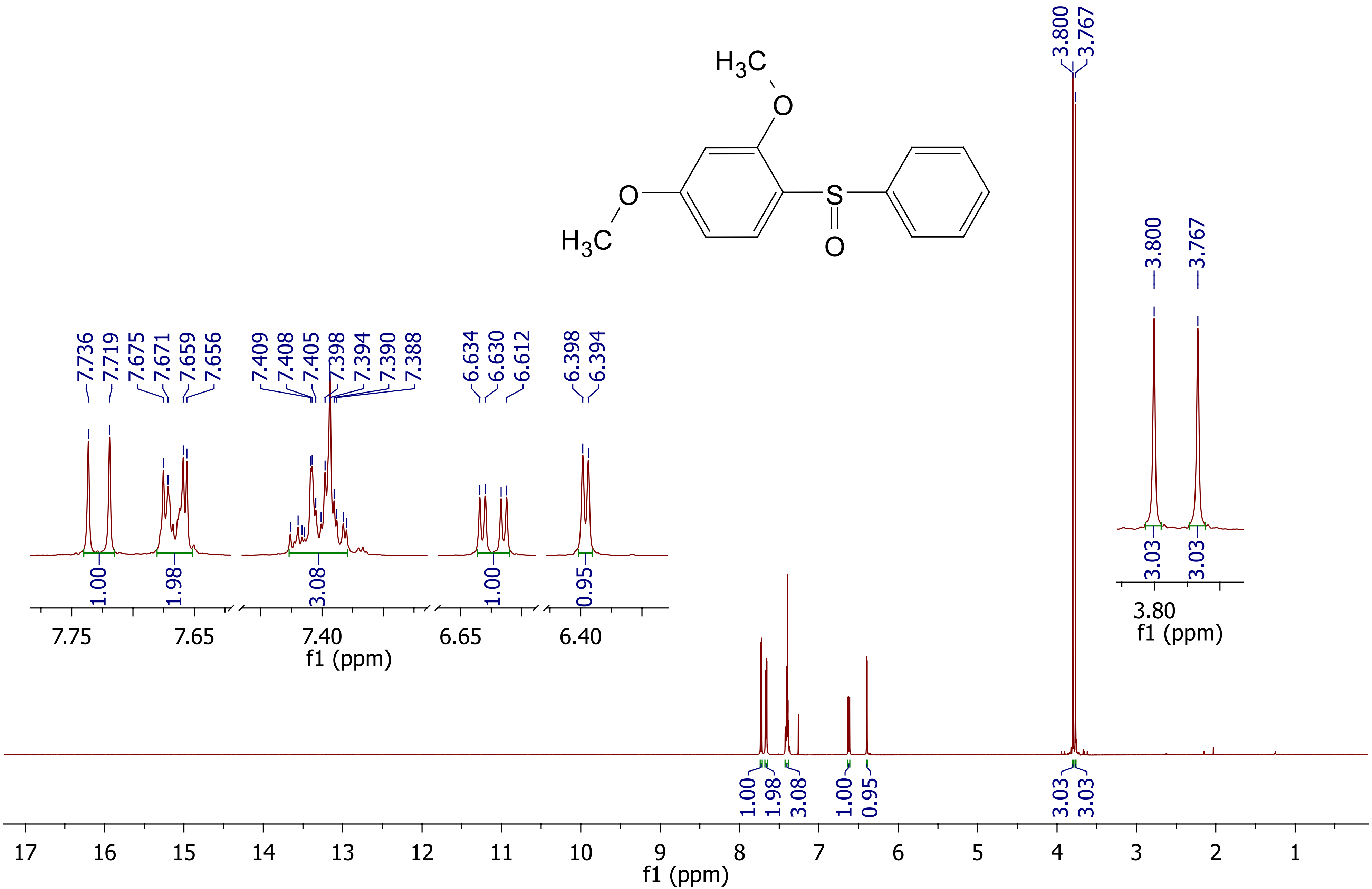

2,4-dimethoxy-1-(phenylsulfinyl)benzene (3a)

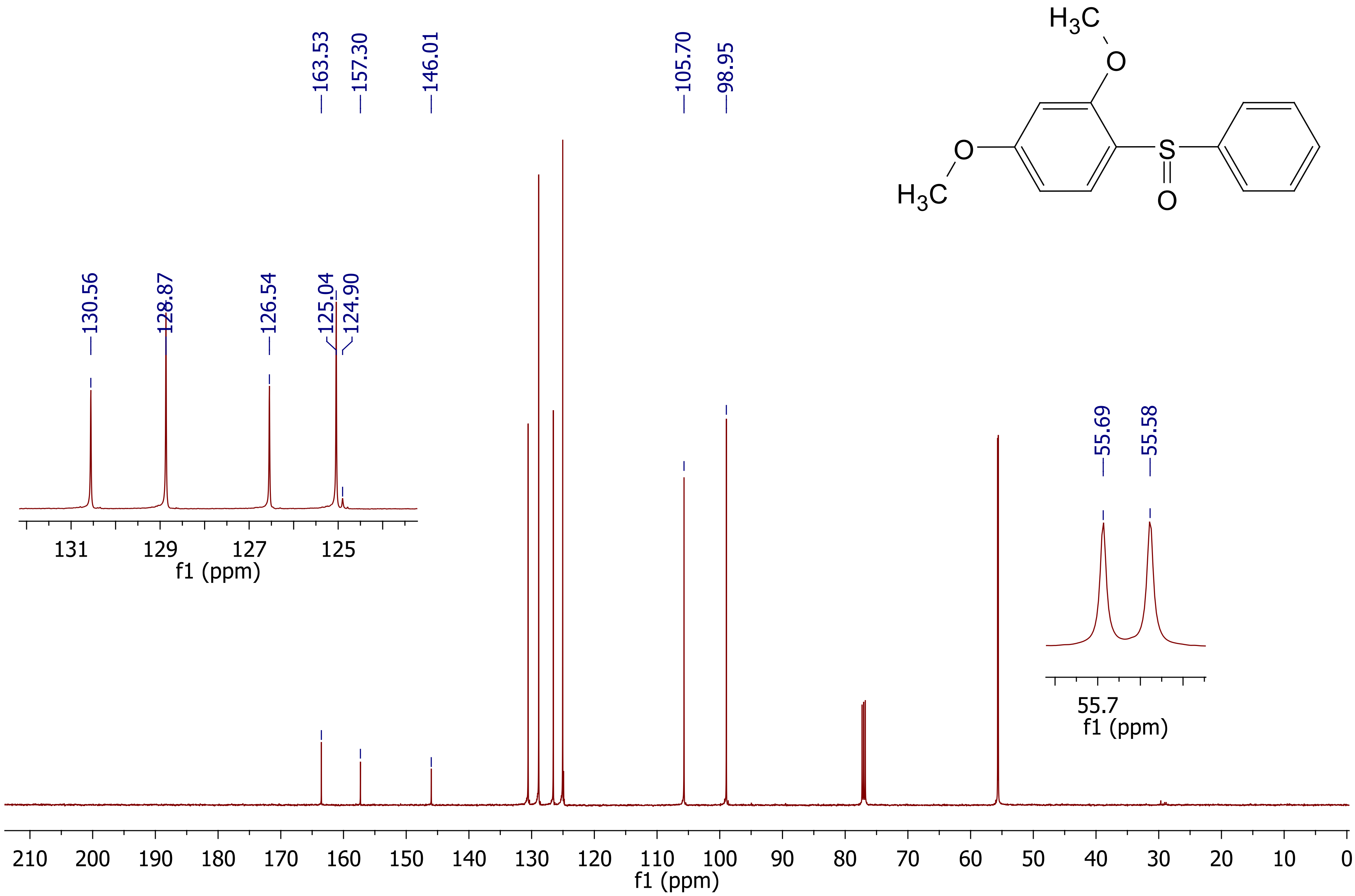

2,4-Dimethoxy-1-(ethylsulfinyl)benzene (3b)

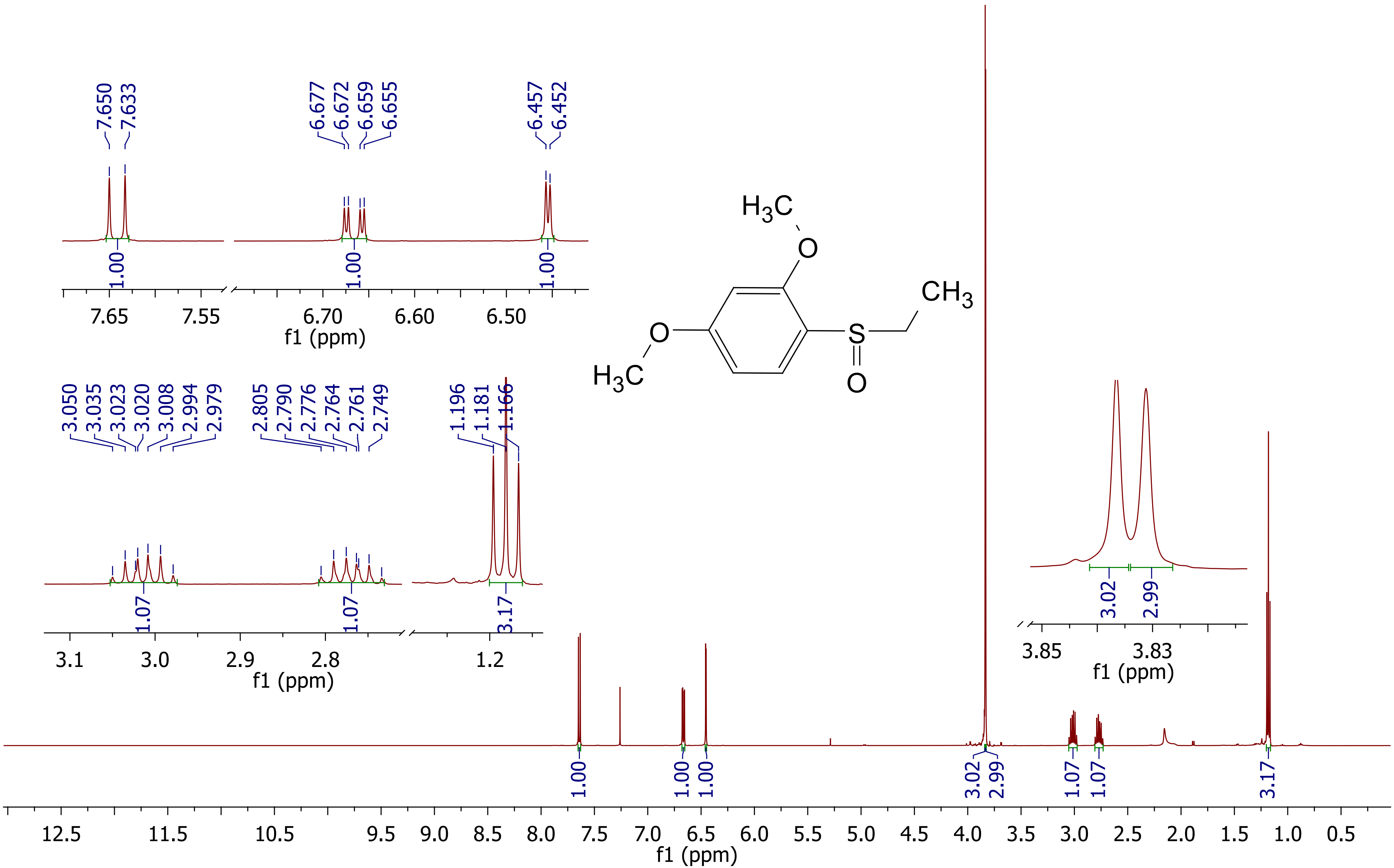

2,4-Dimethoxy-1-(ethylsulfinyl)benzene (3b)

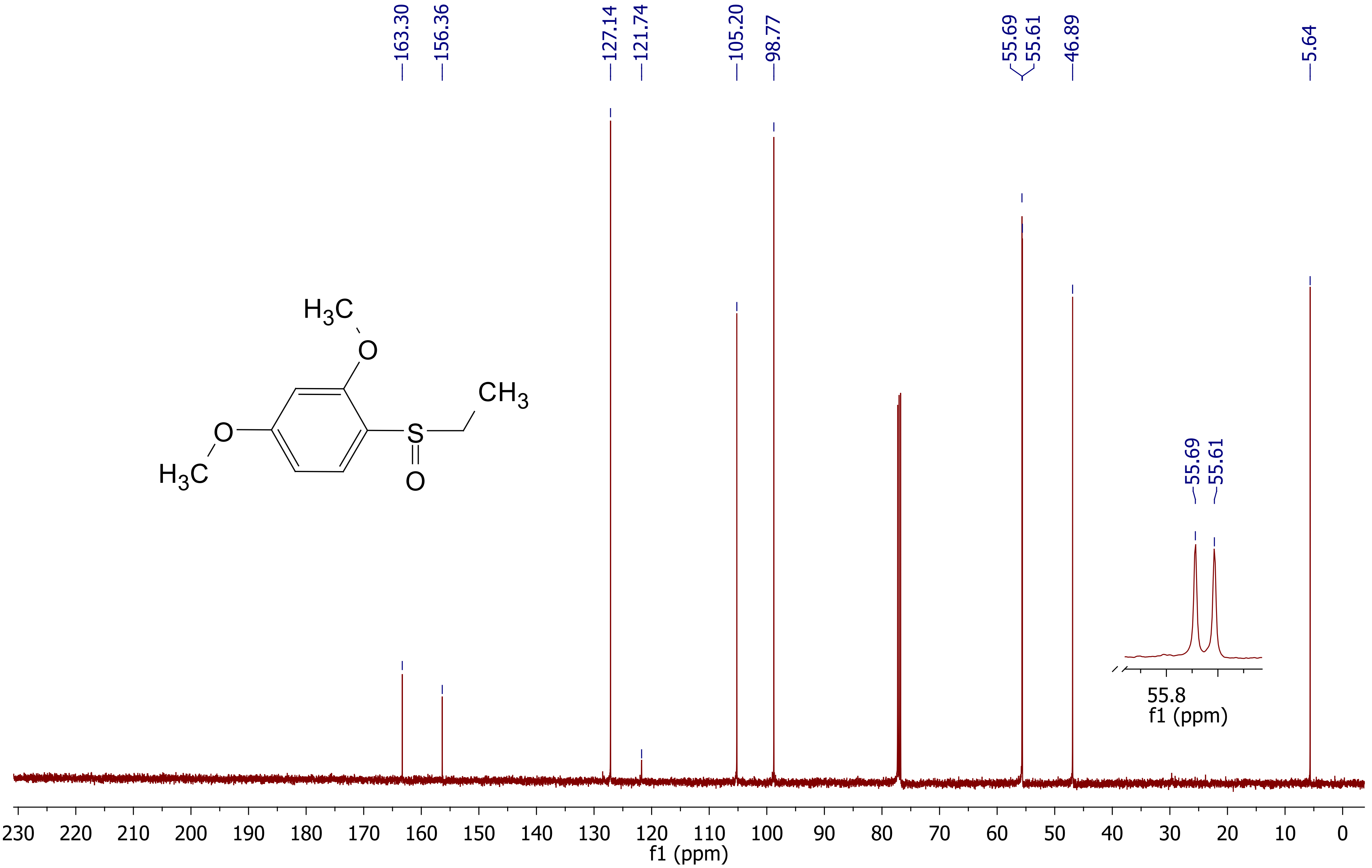

2,4-dimethoxy-1-(2-propylsulfinyl)benzene (3c)

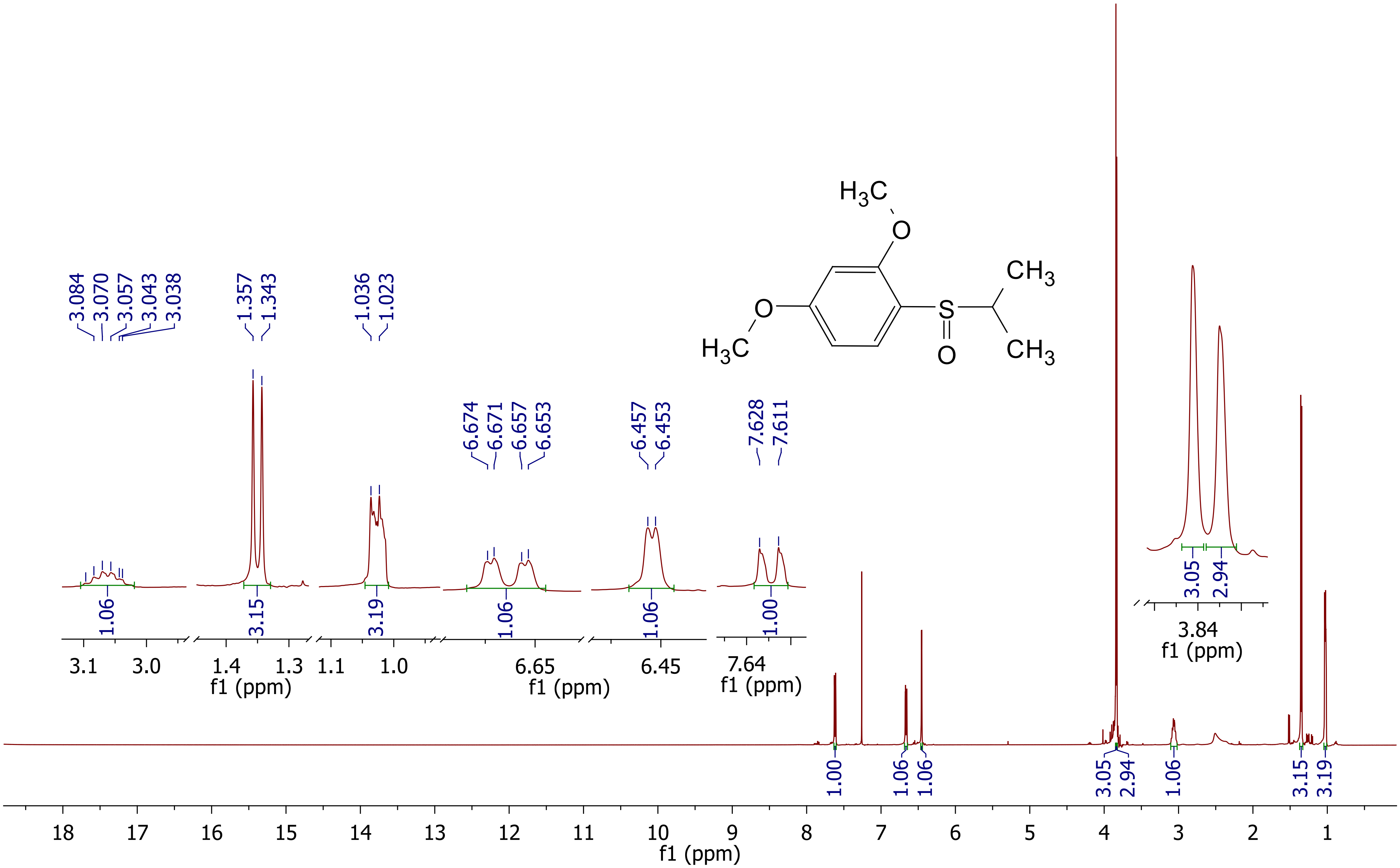

2,4-dimethoxy-1-(2-propylsulfinyl)benzene (3c)

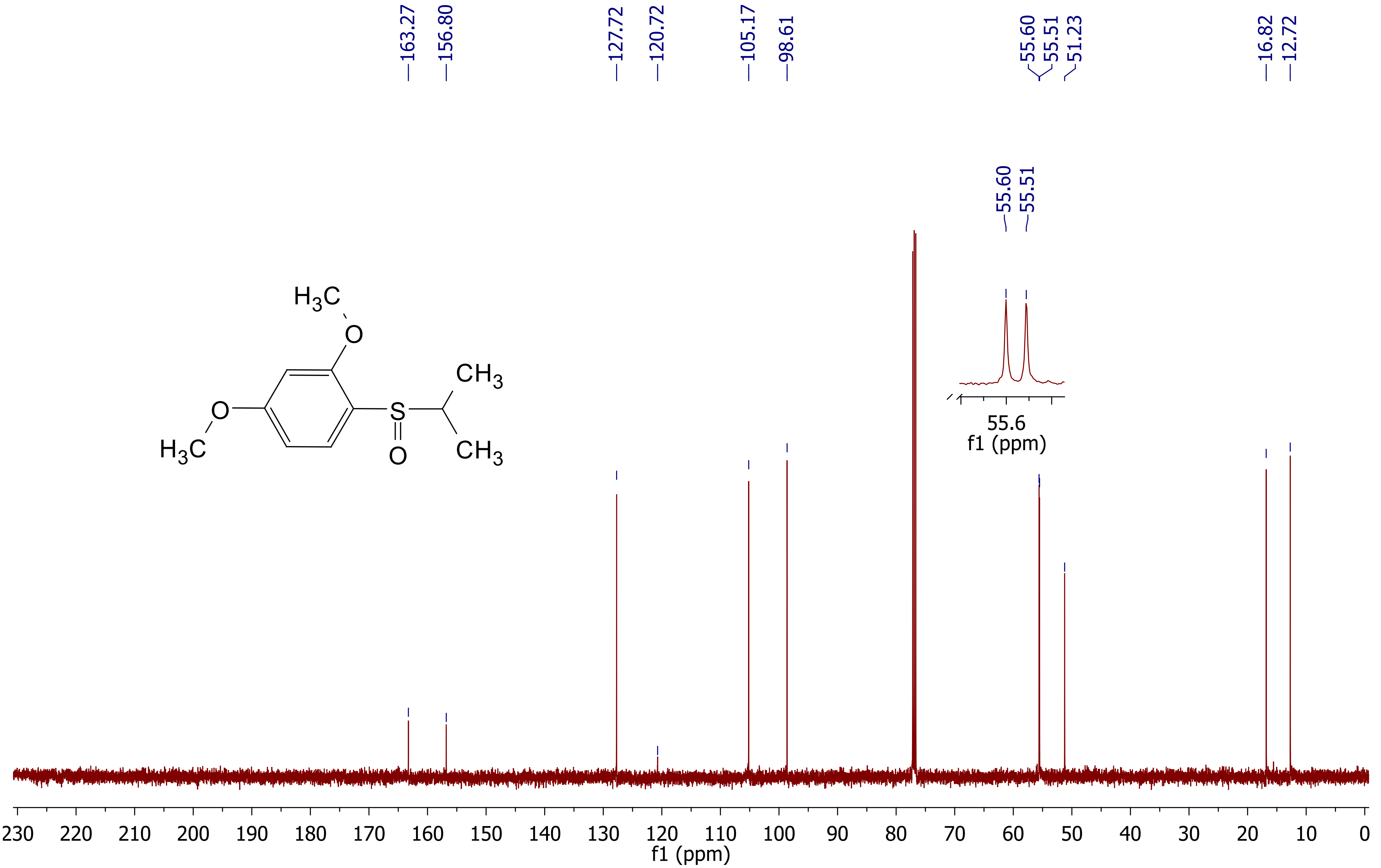

2,4-dimethoxy-1-(octylsulfinyl)benzene (3d)

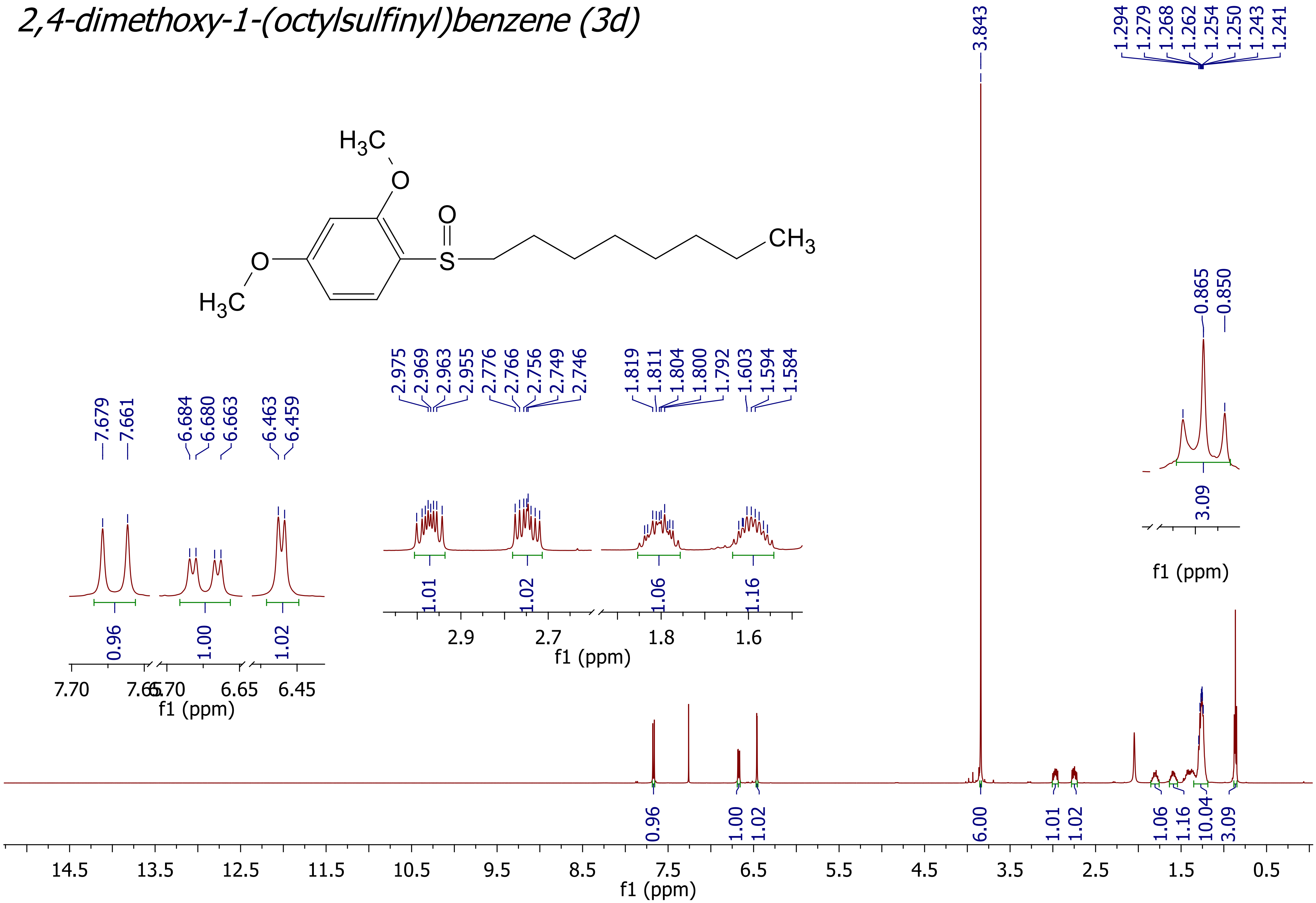

2,4-dimethoxy-1-(octylsulfinyl)benzene (3d)

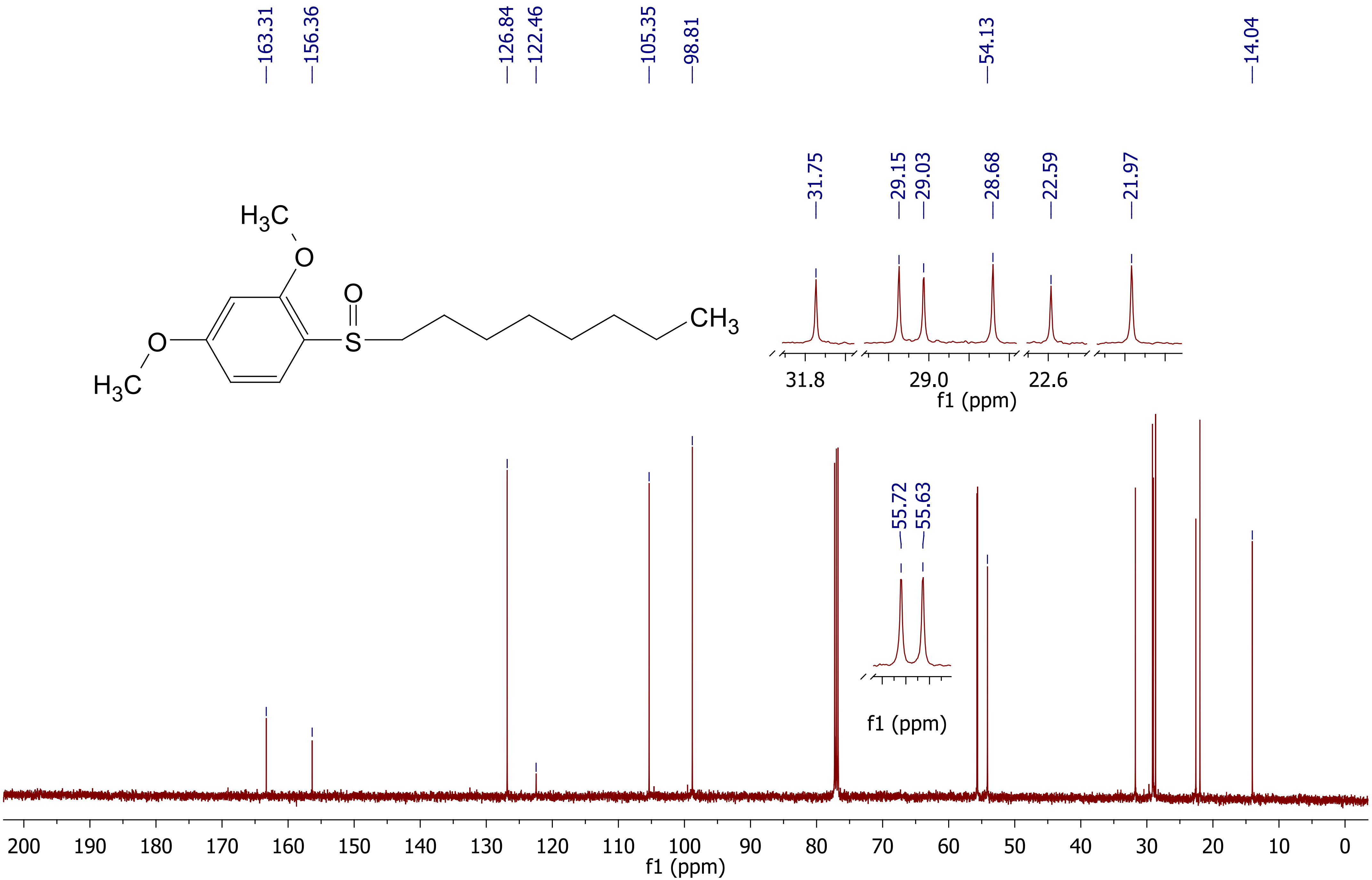

2,4-Dimethoxy-1-(p-tolylsulfinyl)benzene (3e)

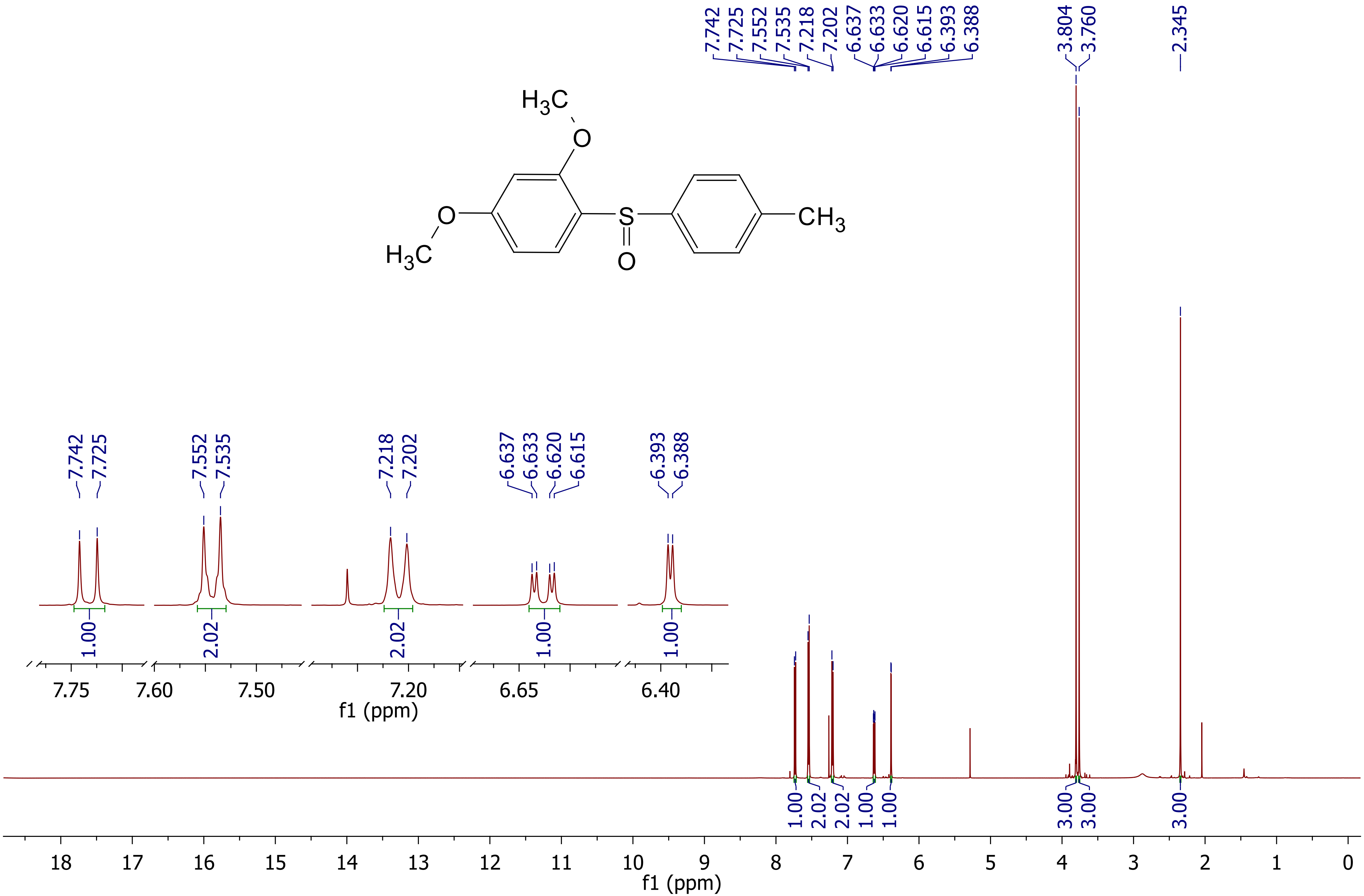

2,4-Dimethoxy-1-(p-tolylsulfinyl)benzene (3e)

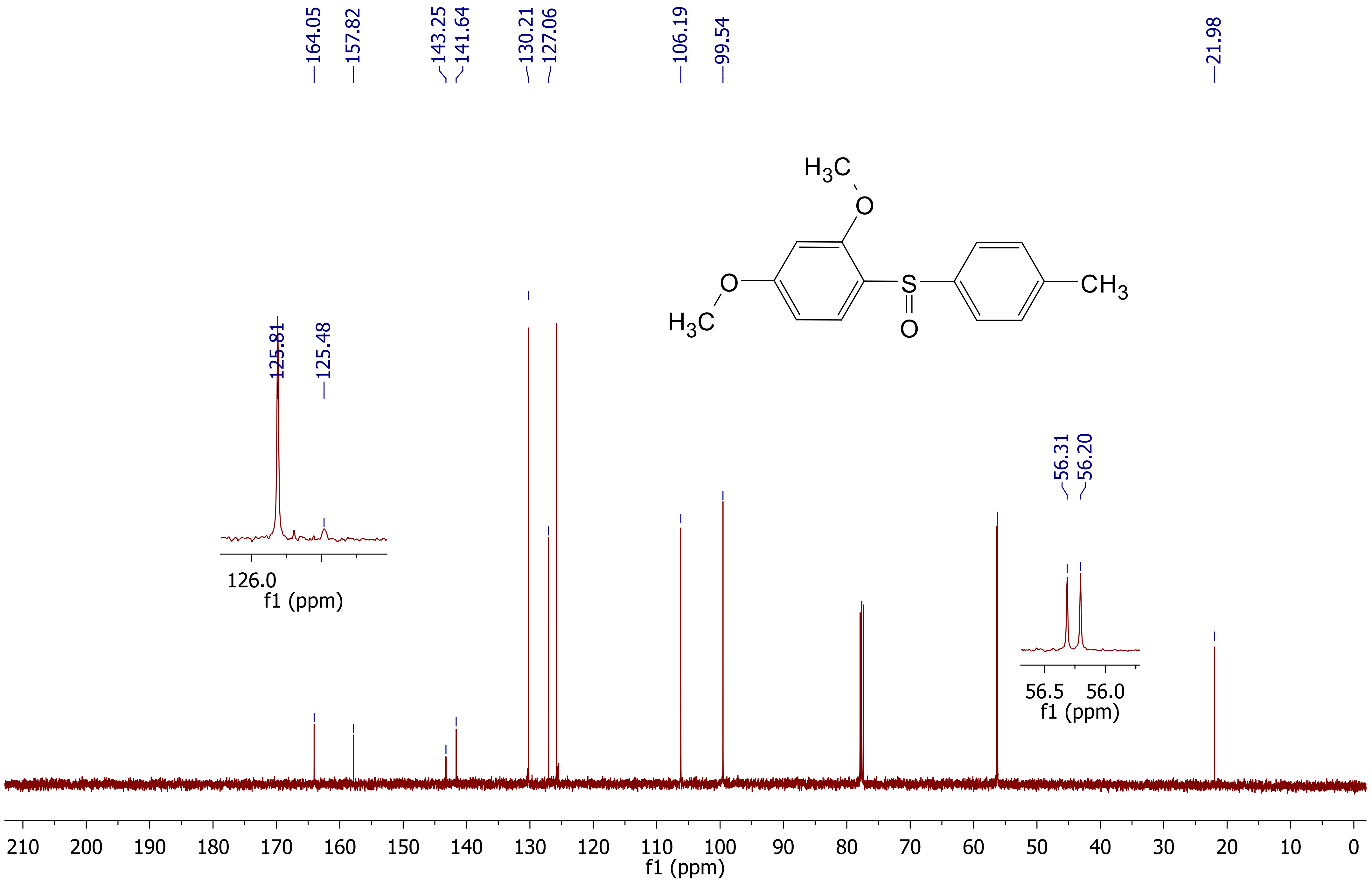

2,4-Dimethoxy-1-((4-methoxyphenyl)sulfinyl)benzene (3f)

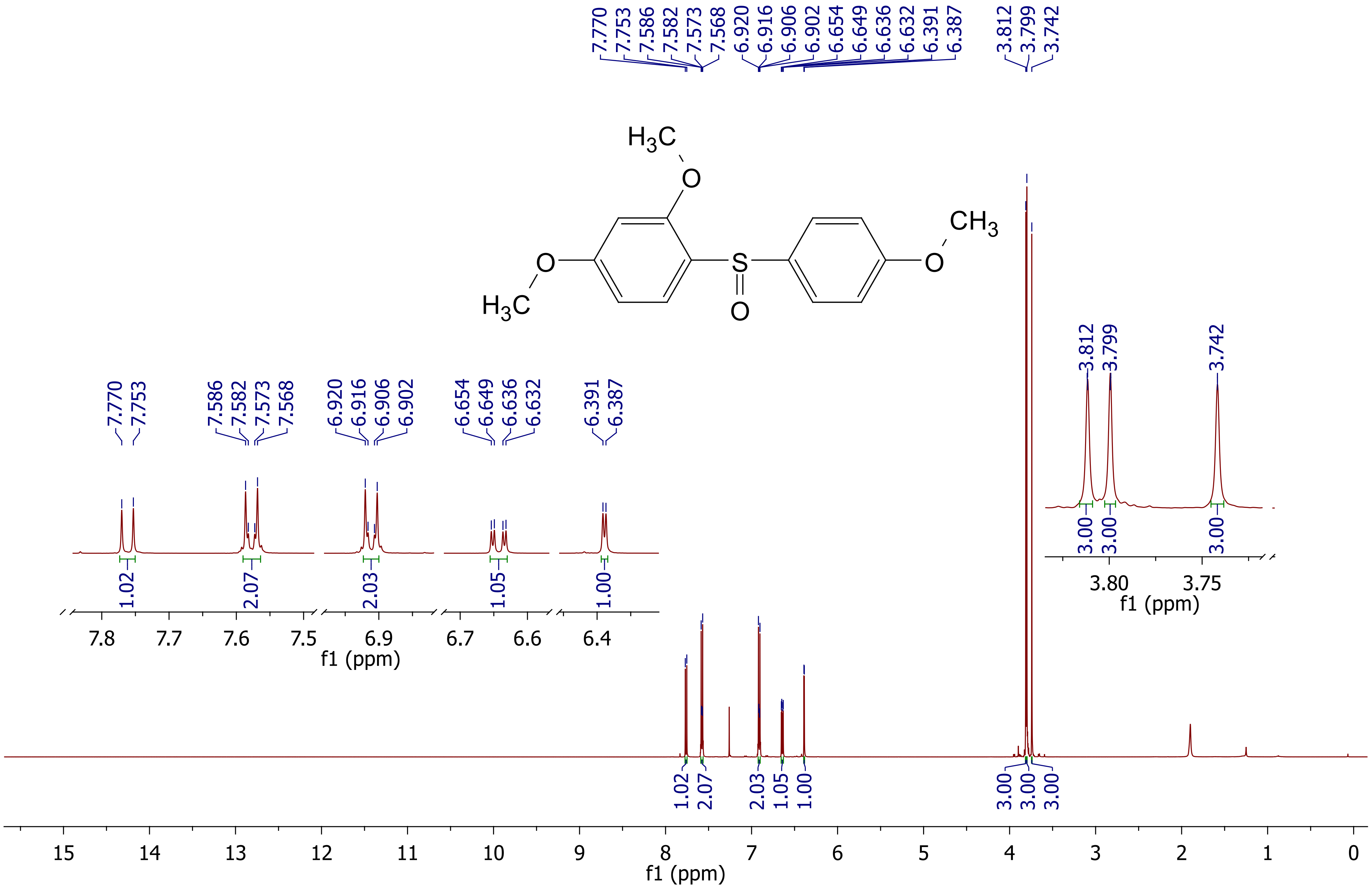

2,4-Dimethoxy-1-((4-methoxyphenyl)sulfinyl)benzene (3f)

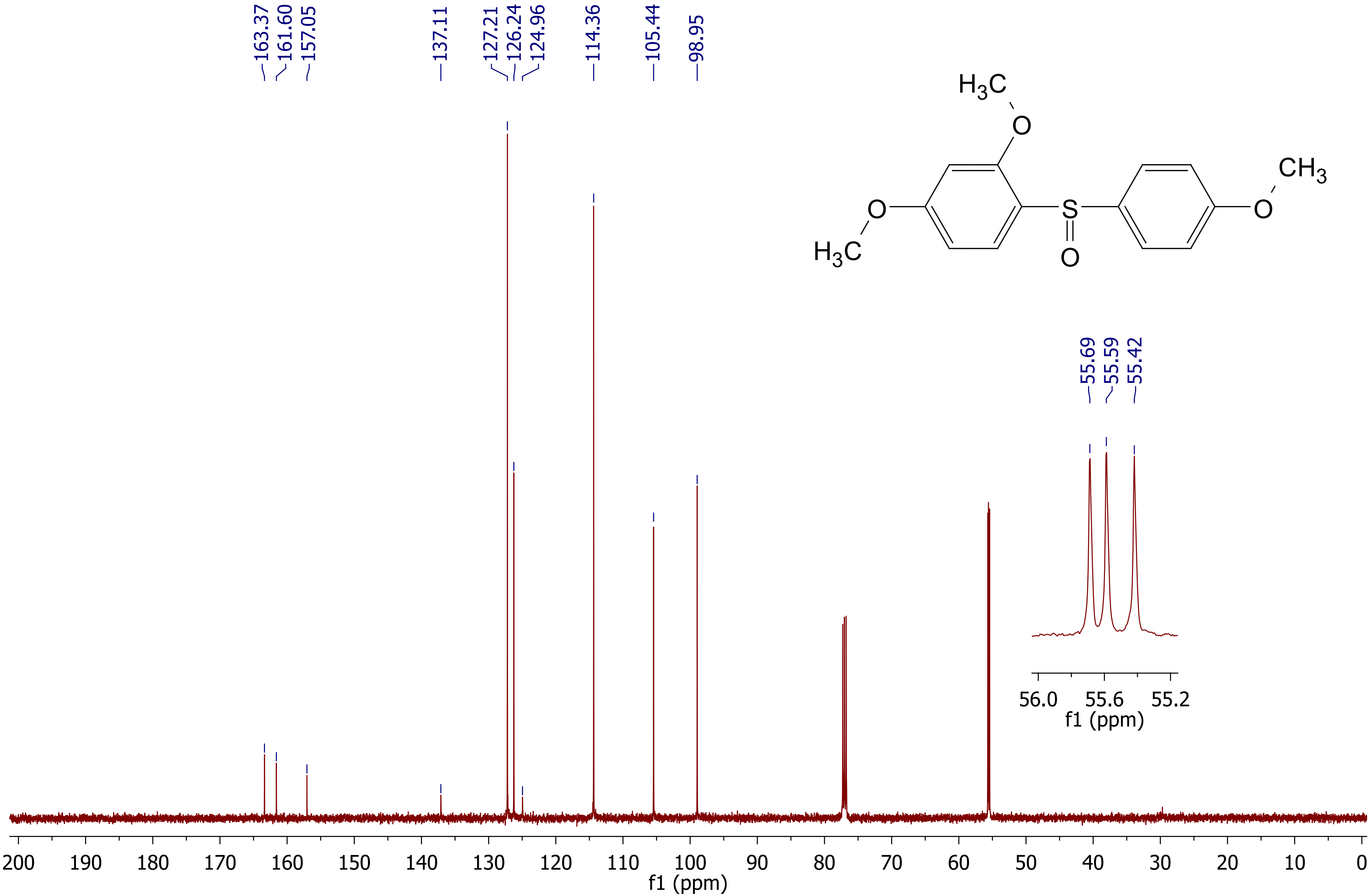

1,2-dimethoxy-4-(phenylsulfinyl)benzene (3h)

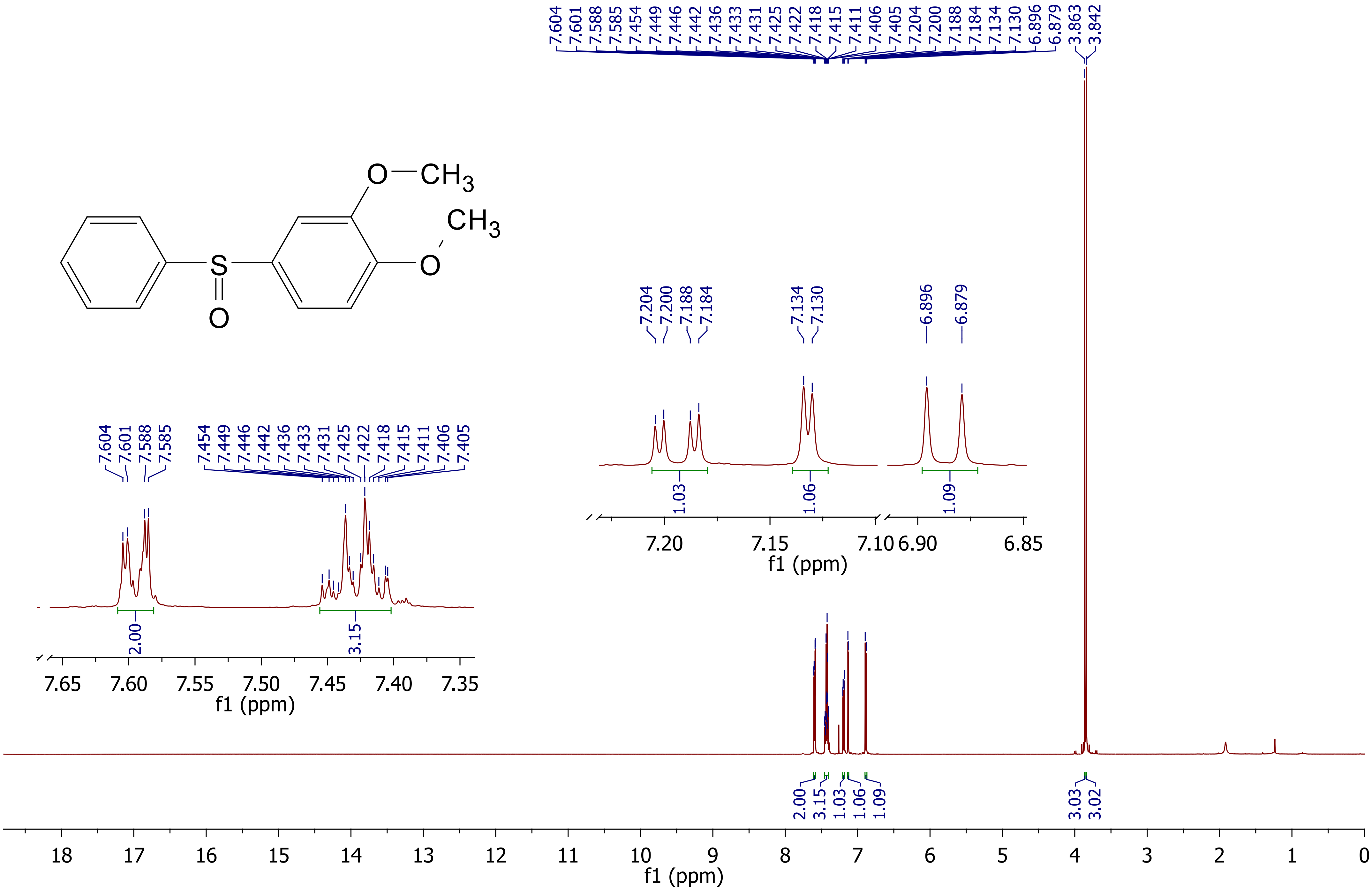

1,2-dimethoxy-4-(phenylsulfinyl)benzene (3h)

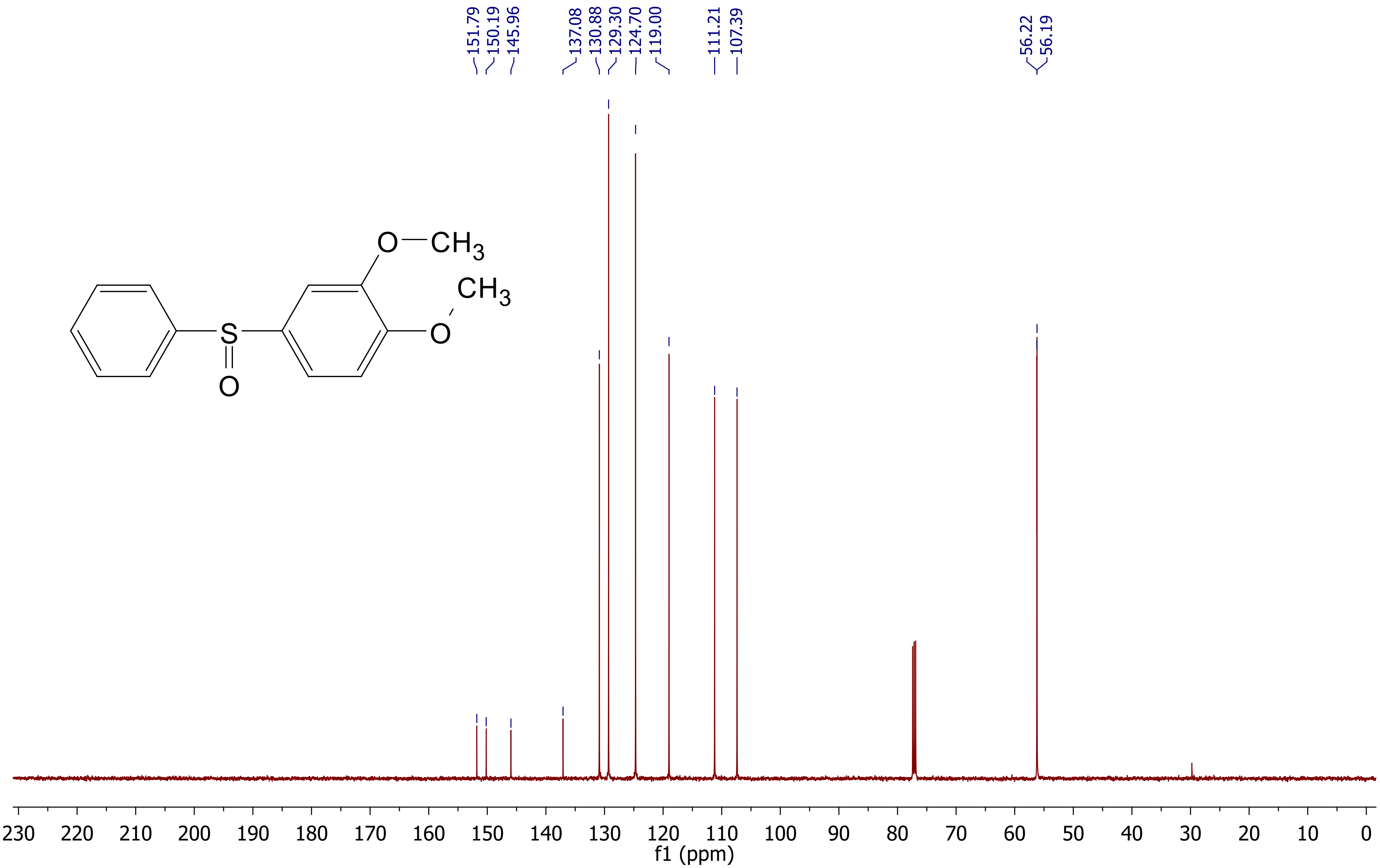

N,N-dimethyl-4-(phenylsulfinyl)aniline (3j)

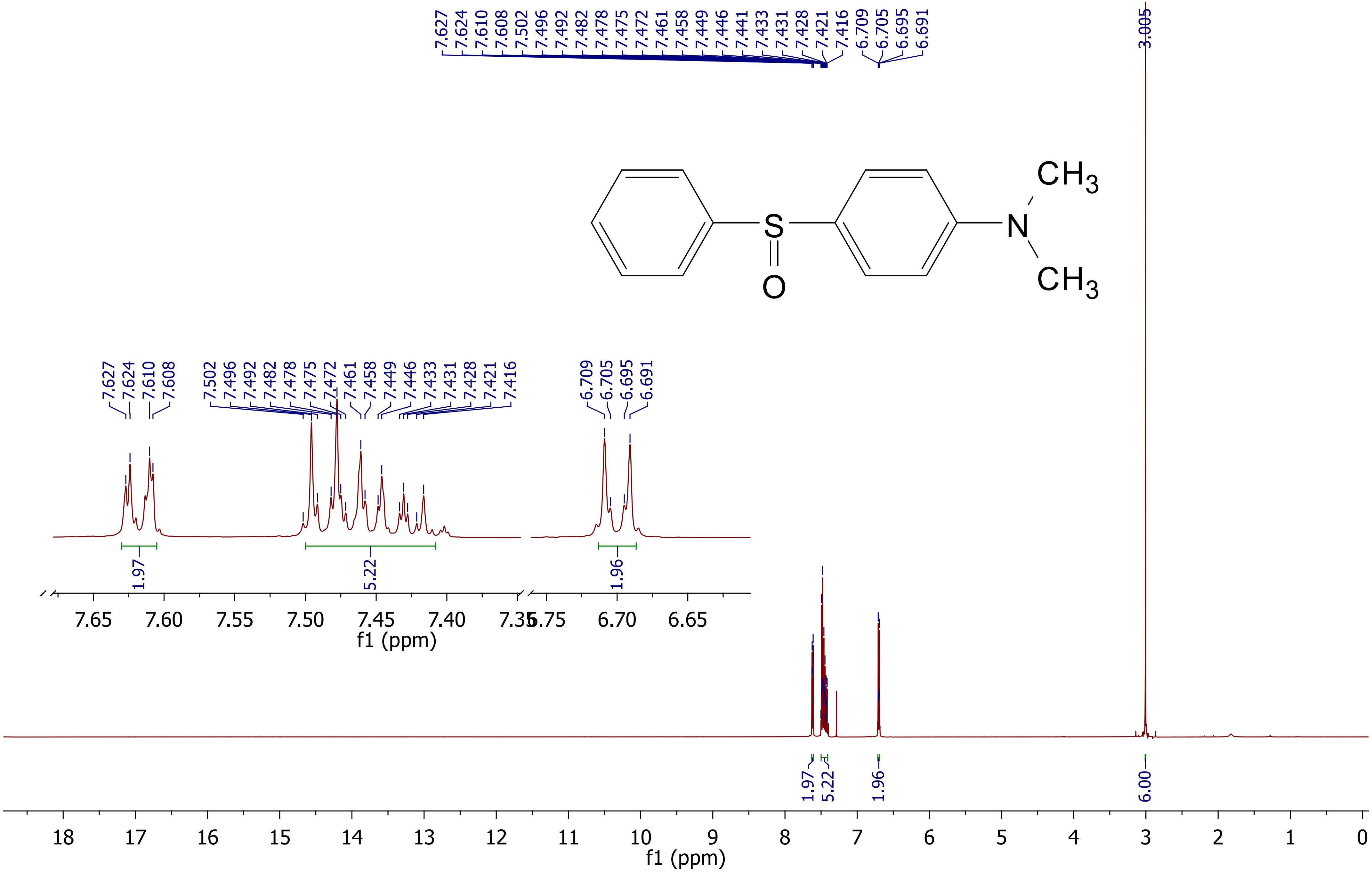

N,N-dimethyl-4-(phenylsulfinyl)aniline (3j)

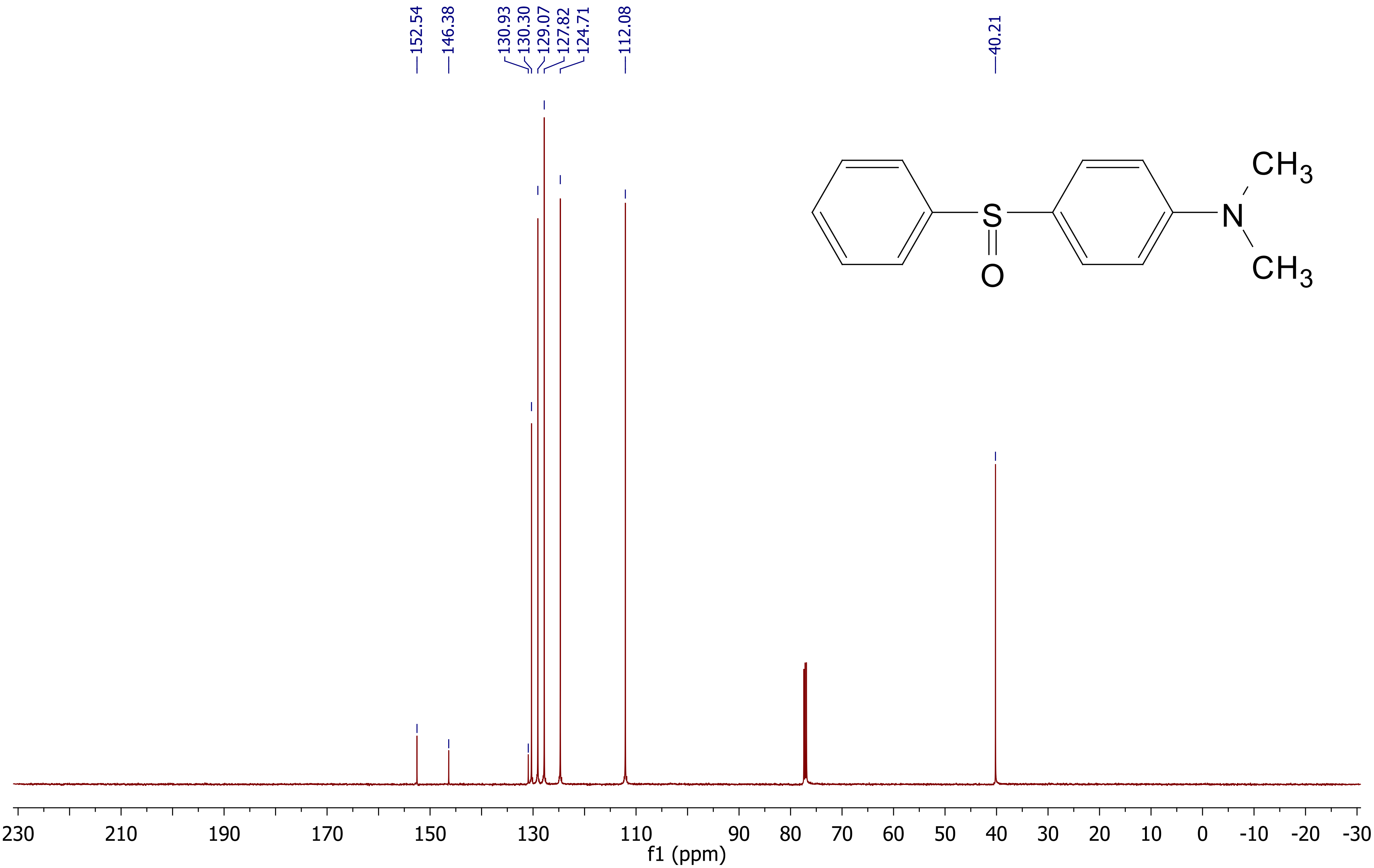

2-methoxy-1-(phenylsulfinyl)napthalene (3l)

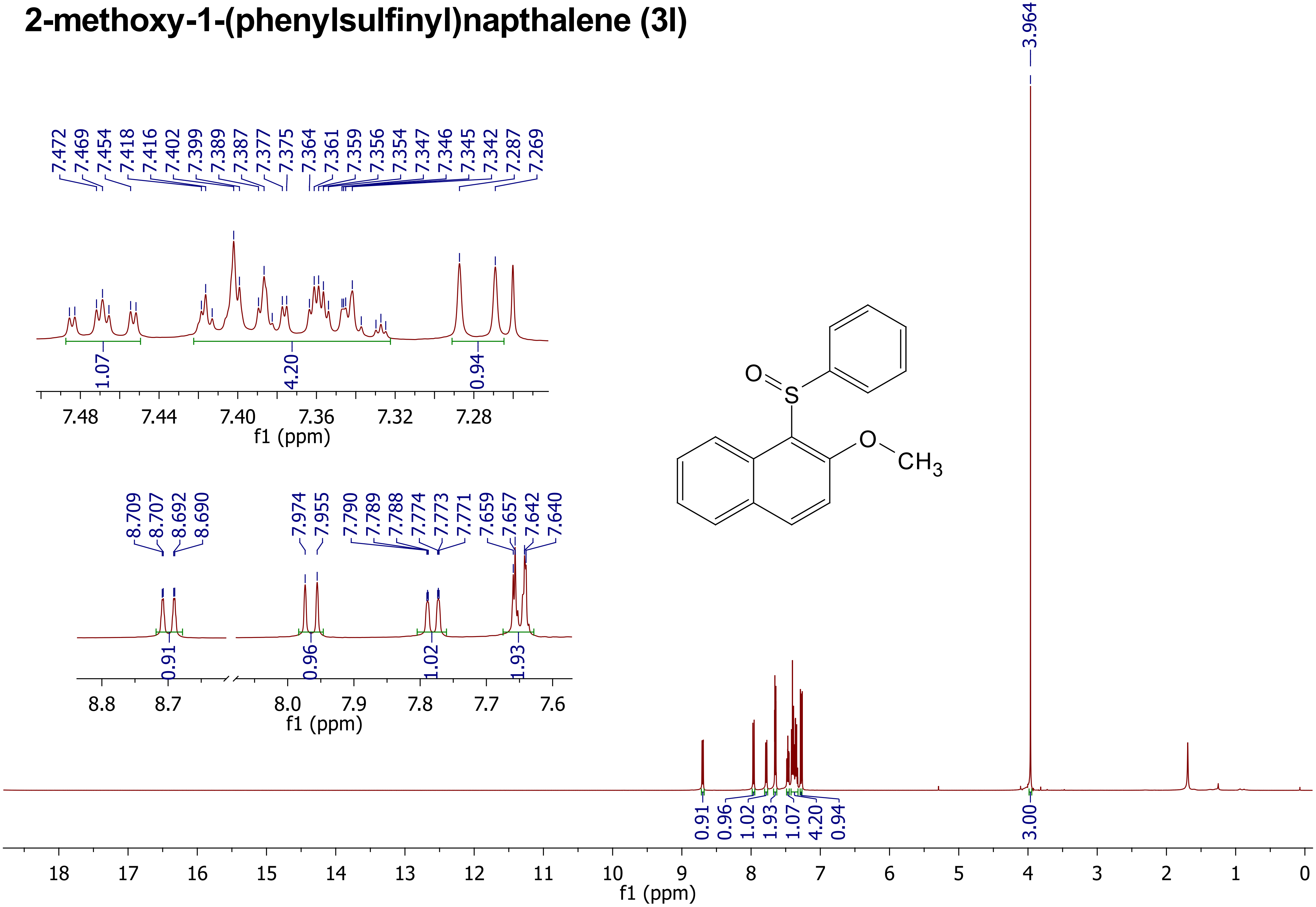

2-methoxy-1-(phenylsulfinyl)napthalene (3l)

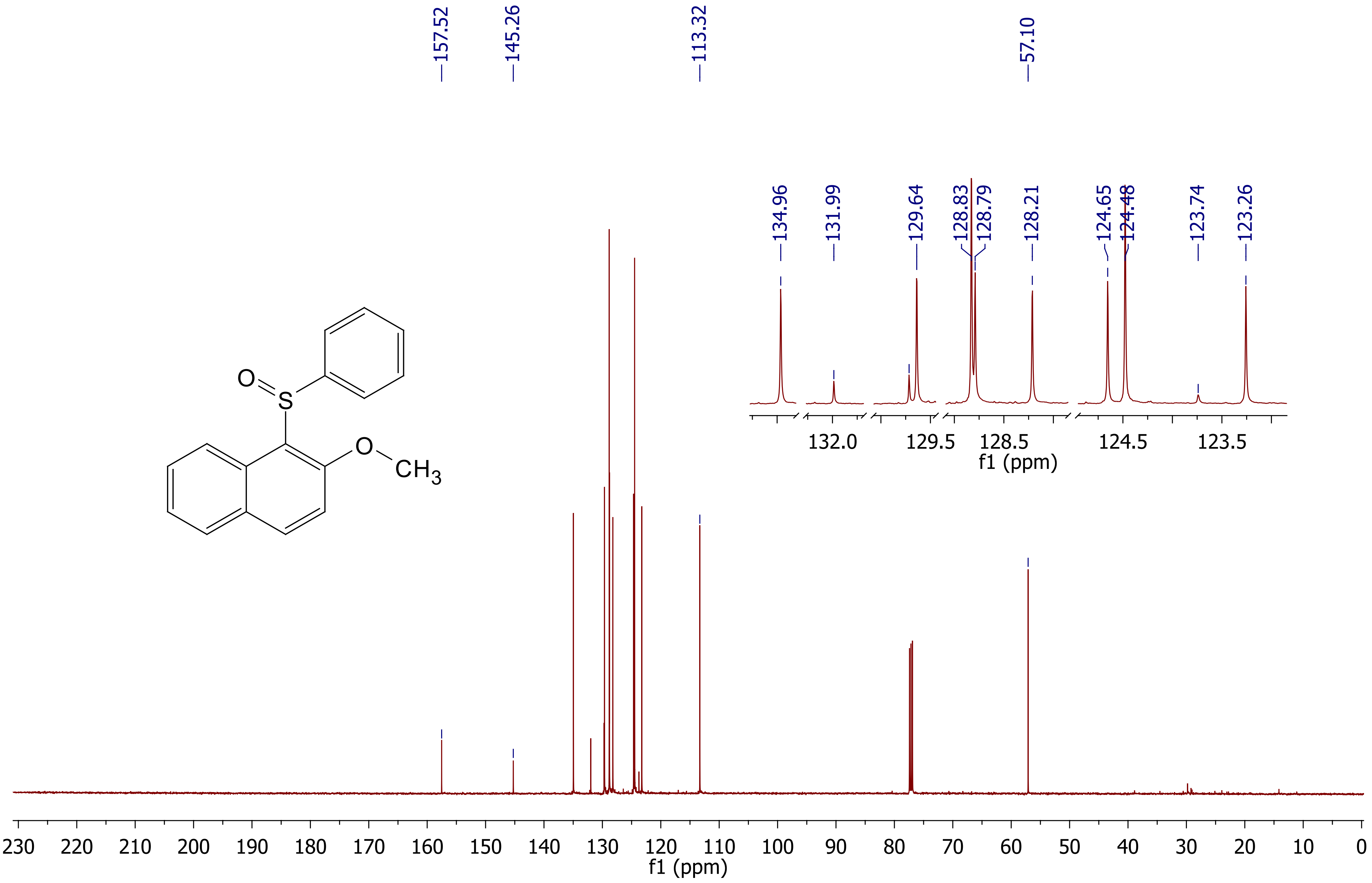

1-methyl-3-(phenylsulfinyl)indole (3m)

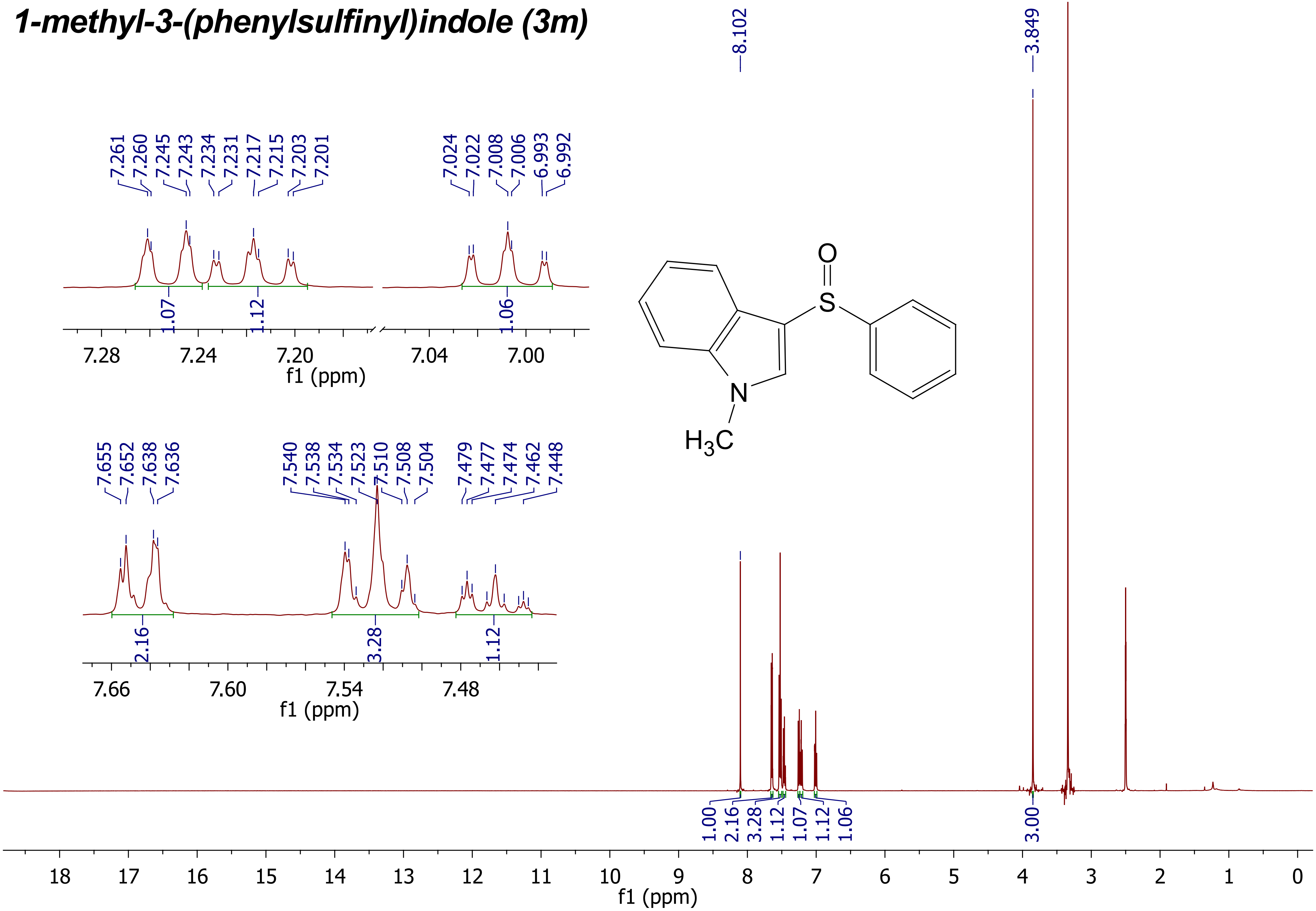

1-methyl-3-(phenylsulfinyl)indole (3m)

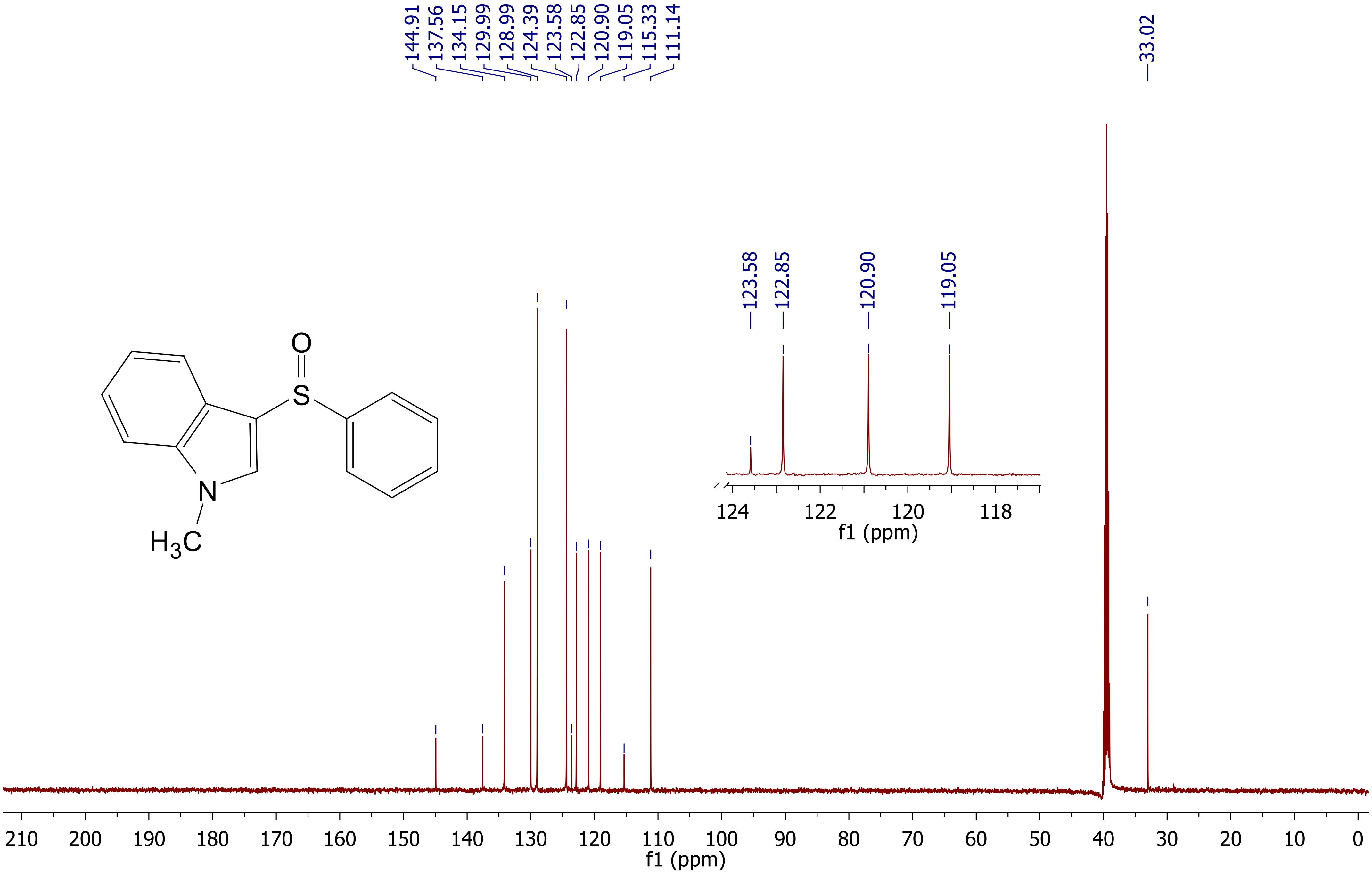

3-(phenylsulfinyl)indole (3n)

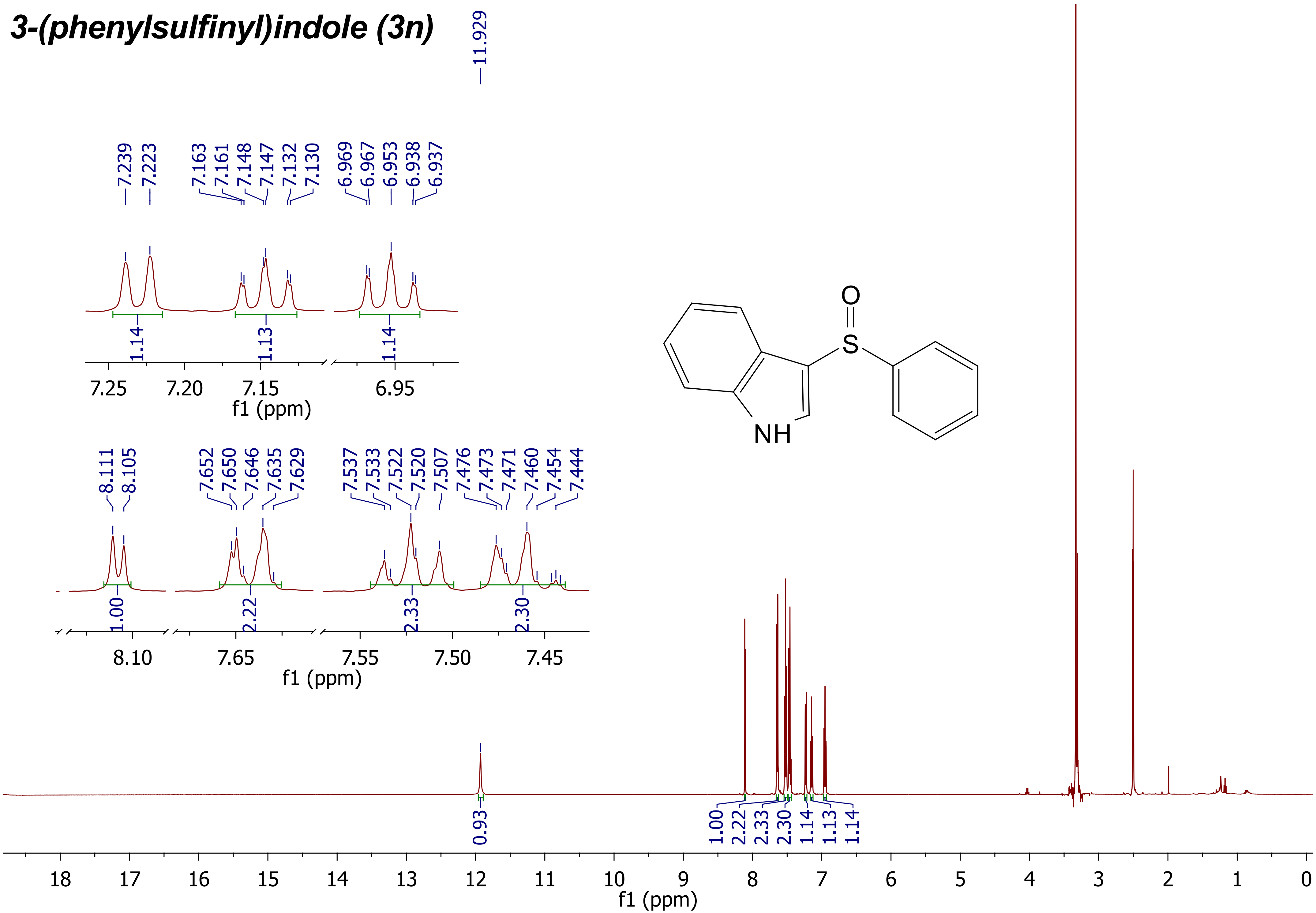

3-(phenylsulfinyl)indole (3n)

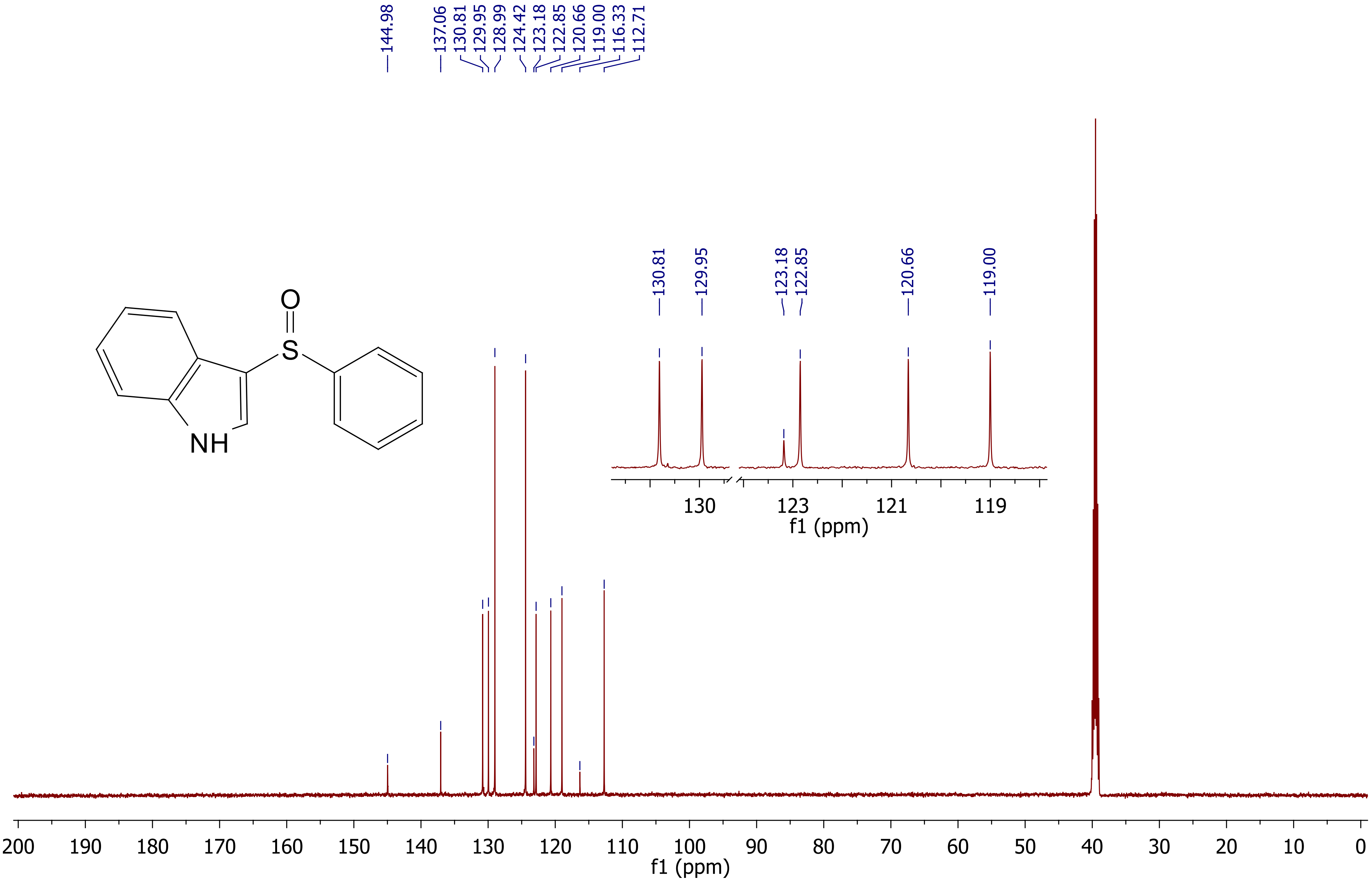

2-(phenylsulfinyl)dibenzo[b,d]thiophene (3p)

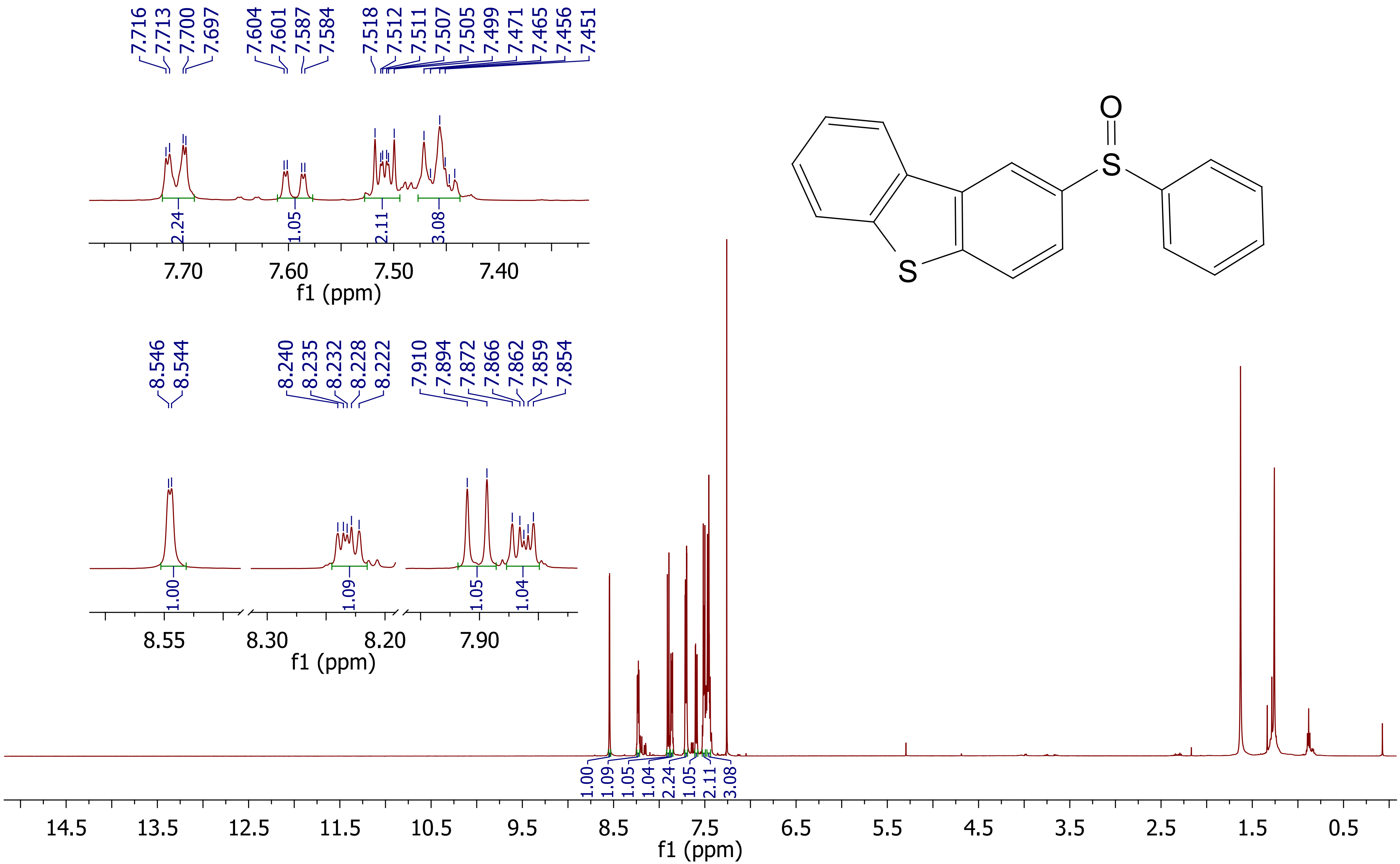

2-(phenylsulfinyl)dibenzo[b,d]thiophene (3p)

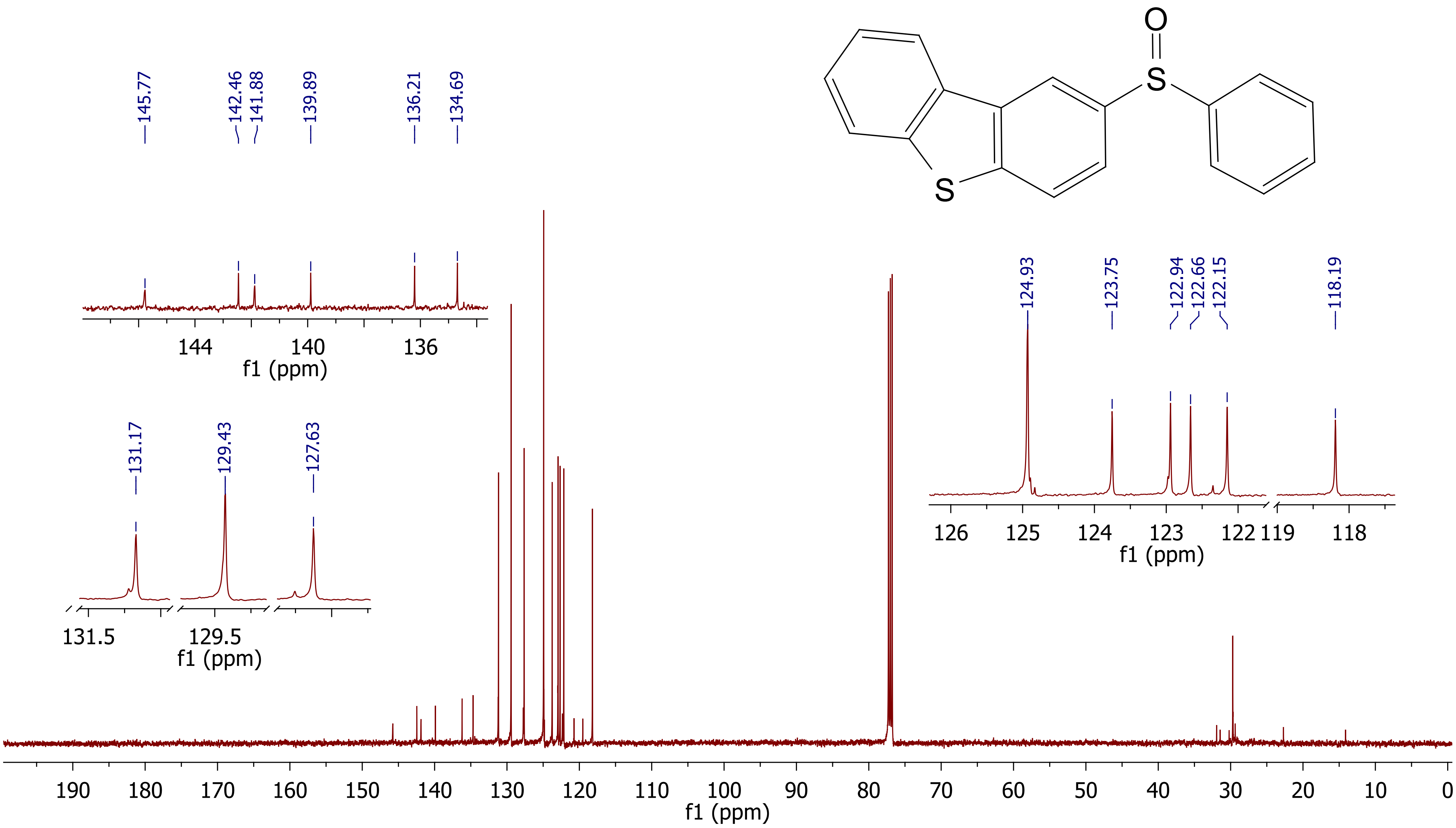

2-(Phenylsulfinyl)dibenzo[b,d]thiophene (3p)

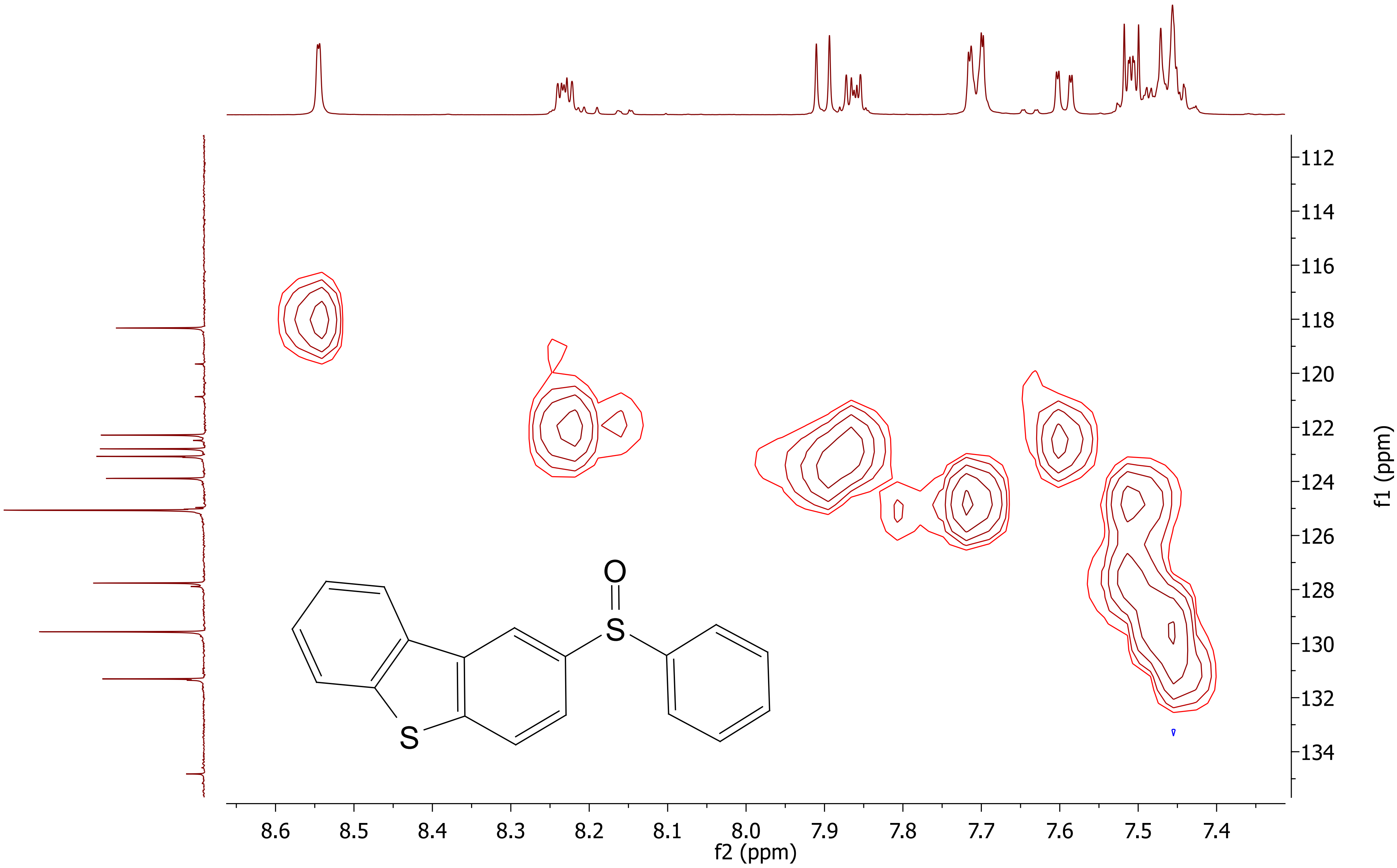

2-(phenylsulfinyl)-9H-fluorene (3q)

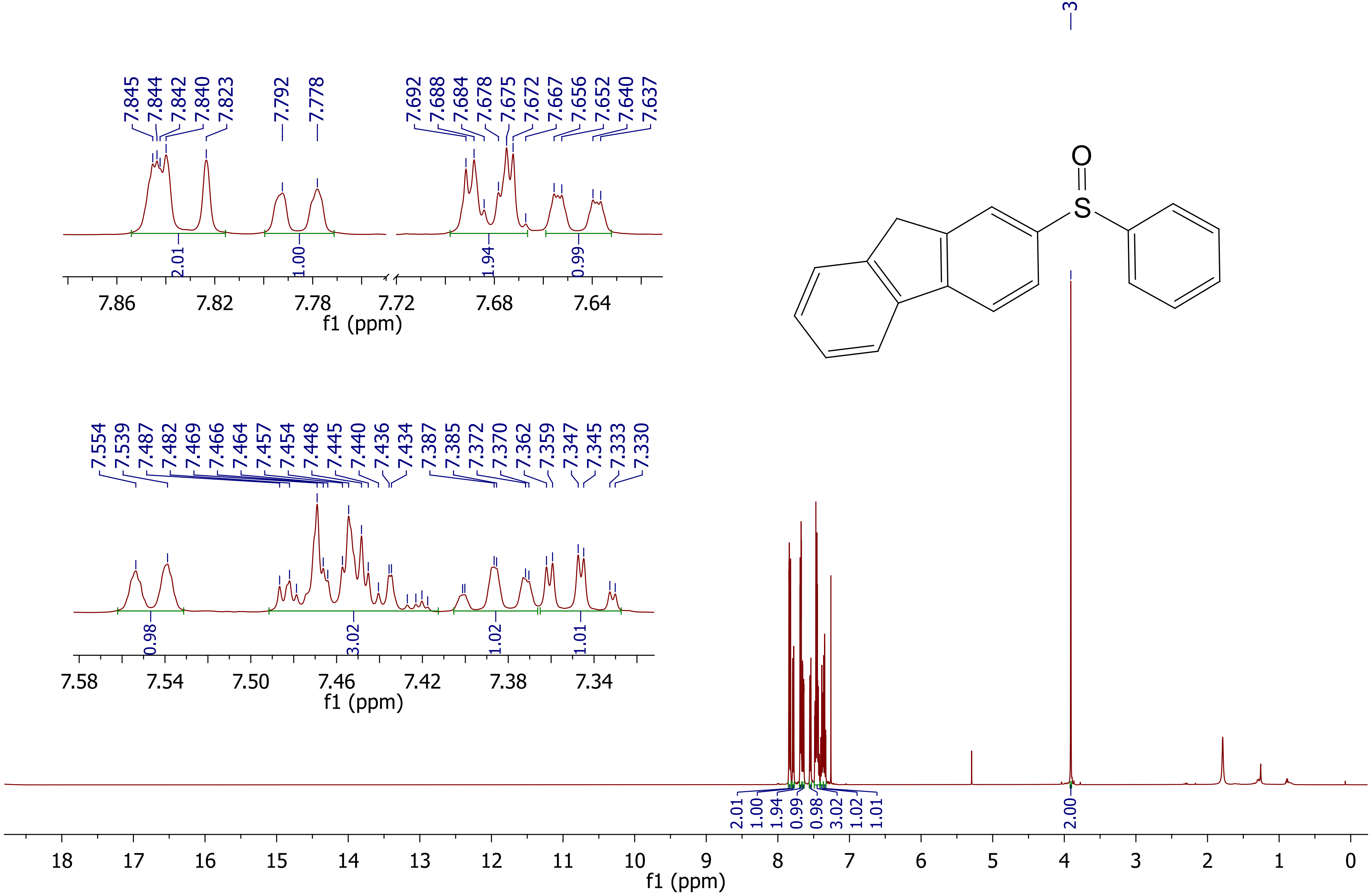

2-(phenylsulfinyl)-9H-fluorene (3q)

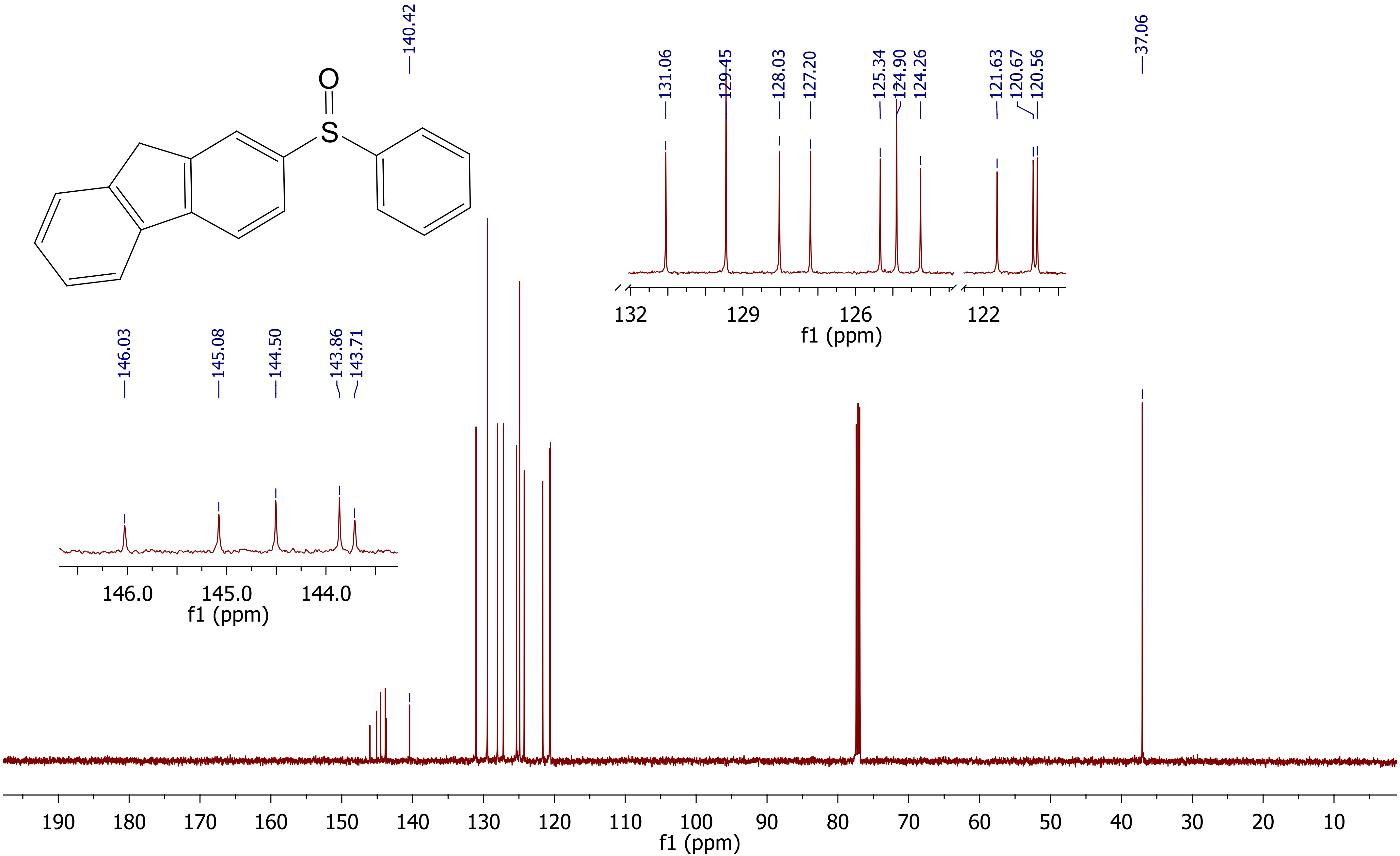

Supplement: Supplementary file 1 [file molecules-22-01458-s001.pdf]
